# LEGO‐like Origami Robots Standardize Structure Design of Soft Robots

**DOI:** 10.1002/advs.202513881

**Published:** 2025-12-12

**Authors:** Zheng Wang, Yuzhe Wang, Hongying Zhang

**Affiliations:** ^1^ Department of Mechanical Engineering National University of Singapore Singapore 117575 Singapore; ^2^ Agency for Science, Technology and Research Singapore Institute of Manufacturing Technology Singapore 138634 Singapore

**Keywords:** modular soft actuators, origami robots, soft robotics

## Abstract

This paper presents a novel design methodology for automatically constructing soft robots with modular, LEGO‐like origami actuators. By assembling these modular soft actuators, intricate robotic systems can be created. The key innovation of the work lies in the incorporation of thickness accommodation into kinematics models and the optimization‐based assembly strategy standardizing the design process. This approach has been validated through two LEGO‐like origami actuators, which are then assembled guided by the assembly strategy. A robot arm built by these actuators showcased a remarkable position accuracy, deviating by only 3 mm at the tip over a 400 mm span. Furthermore, two soft robots are successfully designed, built, and tested for manipulation and tight space inspection, and a bipedal walking robot, confirming the feasibility of the design algorithm for functional robotic systems. This work offers a comprehensive design framework that standardizes the assembly of LEGO‐like origami actuators into accurate and functional robotic systems.

## Graphical abstract

The paper proposes a comprehensive design framework that standardizes the soft robot development process as the assembly of LEGO‐like, off‐the‐shelf origami actuator modules. With actuators designed to accommodate thickness and an optimization‐based assembly strategy, it establishes a repeatable, systematic workflow that streamlines and accelerates building complex soft robotic systems from reusable components.

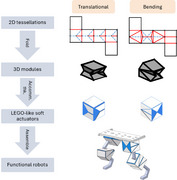

## 1 Introduction

Soft robotics has garnered significant interest among robotic engineers and researchers due to its unique capabilities,^[^ ^1^, ^2^, ^3^ ^]^ including inherent safety to interact with unstructured environments. Successful soft robotic designs include soft actuators,^[^ ^4^, ^5^ ^]^ soft grippers,^[^ ^6^, ^7^, ^8^ ^]^ and soft robotic arms.^[^ ^9^, ^10^ ^]^ Notwithstanding this growing interest, there is a noticeable lack of reports that standardize soft actuators in a way that can be considered modularized and off‐the‐shelf devices, similar to their rigid counterparts. This lack of standardization complicates the design and integration of soft robotic systems, hindering their widespread adoption and scalability.^[^ ^11^ ^]^ Furthermore, the majority of soft robots rely on stretching‐dominated deformation to achieve function. Due to the hyper‐elasticity of the constituent soft materials, conventional soft robots often result in large hysteresis, low position accuracy, and limited durability, making it difficult to be applied in practical scenarios.^[^ ^12^, ^13^ ^]^

Over the decades, numerous soft robots have been proposed to automate the design process of soft robots, yet they still face limitations. Early soft robots come from intuitive and bioinspired designs, where designers rely on their knowledge, intuition, and inspiration drawn from biological systems to design the robots. For instance, Wehner et al. designed and fabricated an octopus‐like soft robot,^[^ ^14^ ^]^ Laschi et al. designed an octopus‐inspired soft robot arm,^[^ ^15^ ^]^ and Katzschmann et al. presented a soft robot fish.^[^ ^16^ ^]^ These designs are ad hoc, heavily relying on the inspiration and experience of the designers. Model‐based and structural optimization designs were investigated to overcome the limitations of intuitive and bioinspired designs. For example, Wang et al. presented a line segment‐based model to simulate a fluidic soft actuator and an optimization strategy to identify size parameters.^[^ ^17^ ^]^ Zhang et al. used a topology optimization method to design soft fingers and Chen et al. proposed a geometry–mechanics–optimization integrated approach to design the morphology of soft pneumatic actuators.^[^ ^18^, ^19^ ^]^ However, both methods come with a high computational cost, particularly when finite element analysis and nonlinearities are involved. This high computational cost poses a significant obstacle to the widespread use of these methods in the field of soft robotics.^[^ ^13^ ^]^

To overcome these challenges, standardizing soft robots by drawing inspiration from rigid robotics can be transformative. By decomposing complex deformations into fundamental contraction, twisting, and bending modes, designers can streamline the system‐level design of soft robots into two steps: developing standardized actuators for basic deformation modes and planning their assembly into functional robotic systems based on specific requirements. An ancient but elegant paper art, origami, has been exploited in the field of soft robotics and holds significant potential as a candidate for realizing these modular designs.^[^ ^20^, ^21^, ^22^ ^]^ The fundamental benefit of origami soft robots lies in their ability to emulate kinematic motions observed in rigid robots–namely, translation and bending–while preserving structural compliance and inherent safety that characterize soft robots. Notably, the kinematic motions are generated through localized bending along folds, which holds the potential to overcome the position accuracy in stretching‐dominated soft robots.^[^ ^23^ ^]^ These lead the robots and actuators based on origami folding theory, an integral part of the soft robotics field, e.g., self‐folding machines,^[^ ^24^ ^]^ origami‐inspired artificial muscles,^[^ ^25^, ^26^ ^]^ bioinspired joint,^[^ ^27^ ^]^ and origami robot arms.^[^ ^28^ ^]^

Origami structures have long been employed to build soft actuators. Peano‐HASEL and pouch motors utilize parallel folding‐based origami patterns, but they are not modularized,^[^ ^29^, ^30^ ^]^ and reports on their position accuracy are rare. Although the bistable origami module designs based on the Kresling pattern have demonstrated potential for assembling various robotic systems,^[^ ^31^, ^32^, ^33^ ^]^ these origami actuators lack position accuracy data. Similarly, Mori offers reconfigurable structures but lacks position accuracy for one module.^[^ ^34^ ^]^ Cable‐driven origami actuators inspired by the Yoshimura pattern with kinematic models demonstrated position accuracy of 1.27 and 0.373 mm in one direction under closed‐loop control,^[^ ^35^, ^36^ ^]^ respectively, yet without demonstrating in different robotic systems. Chen et al. proposed an algorithm to generate crease patterns for origami chains.^[^ ^37^ ^]^ However, their use of thin materials limits practical applications, and accuracy has not been experimentally validated (Detailed review and Table S1, Supporting Information).

With the exception of origami chain,^[^ ^37^ ^]^ none of the aforementioned studies developed a standardized design process to the task‐specific assembly of modular origami actuators. Additionally, none of the works incorporate material thickness in the kinematics modeling, a limitation that can affect the motion range and position accuracy of origami actuators, potentially leading to degraded performance and increased design complexity and cost. In summary, while many origami actuators contribute to modular design in soft robotics, most do not comprehensively address key aspects such as standard assembly strategy, thickness accommodation, positional accuracy evaluation, and the demonstrated capability to rebuild diverse robotic systems.

Regarding actuation method, various actuation strategies have been explored for soft and origami robots, including cable‐driven mechanisms,^[^ ^36^, ^38^ ^]^ magnetic actuation,^[^ ^28^, ^39^ ^]^ and smart materials such as shape memory alloys (SMAs),^[^ ^40^ ^]^ liquid crystal elastomers (LCEs),^[^ ^41^ ^]^ and dielectric elastomers.^[^ ^42^ ^]^ Each of these approaches, however, presents notable limitations. Cable‐driven actuators require rewiring and recalibration of cable lengths whenever modular actuators are reassembled, adding considerable effort and reducing modularity. Magnets enable wireless, untethered actuation. However, the requirement for external magnetic field generators poses challenges for scalability and results in complex experimental setups. SMAs and LCEs can generate large stresses and deformations upon heating but suffer from slow cycle times, hysteresis, and limited long‐term durability, restricting their applicability in tasks requiring rapid and repetitive motions. Dielectric elastomers offer high efficiency, compliance, and the ability to achieve large deformations, but their reliance on high external voltages raises safety concerns and integration challenges, limiting their practicality in many robotic systems. Pneumatic actuation is one of the most widely adopted approaches in soft robotics due to its distinct advantages. Pneumatic actuators are capable of producing large, reversible deformations with fast response times, making them particularly well‐suited for applications that demand adaptability and repeatability.^[^ ^25^, ^33^, ^43^ ^]^ Furthermore, some recent works have already been developed to reduce the complexity of pneumatic circuits.^[^ ^44^, ^45^ ^]^ For these reasons, pneumatic actuation was employed in this work.

We have designed modular origami actuators for translation, bending, and twisting, and demonstrated their possibility to assemble into complex soft robots.^[^ ^46^ ^]^ In this work, building upon the foundational capabilities of modular origami constructs, we propose an innovative optimization‐based assembly strategy to streamline the design of robotic systems composed of modular LEGO‐like origami soft actuators. These actuators are uniquely designed to accommodate material thickness and are capable of high‐accuracy bending and contraction motions, owing to their folding‐driven kinematics. Our investigation delves into the geometric analysis of these modular soft actuators and integrates their material thickness into the kinematic modeling to achieve superior position accuracy during the assembly of complex robotic systems. The key symbols are listed in Table S2 (Supporting Information). The optimization process is tailored to minimize the distance between the base and endpoint while ensuring the motion range of each individual LEGO‐like soft actuator is satisfied and pre‐known obstacles are circumvented.

To validate the effectiveness of our assembly and thickness accommodation strategy, we fabricate prototypes using 3D printing and the lamination technique.^[^ ^46^, ^47^ ^]^ Experiments assessed the blocked force at various folding rates and the durability of the LEGO‐like origami actuators, which generated peak forces of around 80 N for translational actuators and 70 N for bending actuators, withstanding 10 000‐cycle of durability tests. We developed a robot arm to test the efficiency of the assembly strategy and the thickness accommodation approach. The accuracy of the robot arm reaches around 3 mm positional discrepancy at the endpoint over a 400 mm span. To further verify the adaptability of our optimization‐based design method in scenarios involving obstacles and large angles along the trajectory, we developed an obstacle‐avoiding robotic arm. Soft robots offer distinct advantages for confined space inspection due to their flexibility and reconfigurability. Additionally, their ability to absorb shock energy from collisions with the environment also makes tfhem ideal for locomotive tasks. To demonstrate the practical utility of the LEGO‐like origami actuators, we implemented a sequential actuation detecting robot arm and a bipedal walking robot. These case studies underscore the adaptability and skill of actuators, as well as the capacity of our methodology to standardize the design process of soft robots.

## 2 Results

This work marks a notable step forward in the field of soft robotics by developing LEGO‐like modularized soft actuators and offering a comprehensive design framework for the systematic design and assembly of complex robotic systems. Alike LEGO toys, the soft robot design framework also includes two crucial tasks: modularized actuators and an assembly map. Rigorous experimental verification of the design methodology, along with a characterization of the mechanical performance of the LEGO‐like soft origami actuators. To demonstrate the effectiveness of our optimization‐based assembly strategy and highlight the versatility of the origami actuators, we successfully developed a placing manipulator, an inspection manipulator, and a bipedal robot.

### 2.1 LEGO‐Like Origami Actuators

#### 2.1.1 Design of Basic Folding Pattern

In the realm of rigid robotics, the deconstruction of complex kinematic motions into their fundamental prismatic and rotational components has been pivotal for quick advancements and innovations. This modular approach has been instrumental in simplifying the construction and enhancing the versatility of rigid robotic systems. Applying a similar philosophy to soft robotics has the potential to significantly streamline the assembly and design processes of these systems. By using a LEGO‐like methodology to create modular components capable of specific movements, we can construct more complex soft robotic systems in a more efficient and scalable manner. The kinematic behavior of soft robots, predominantly characterized by bending and translational movements, contrasts with the rotational and prismatic movements seen in their rigid counterparts. In our preliminary work, we developed two distinct types of LEGO‐like origami actuators, each designed to fulfill one of these primary motions: translational and bending motion, respectively.^[^ ^46^ ^]^

**Figure 1:**
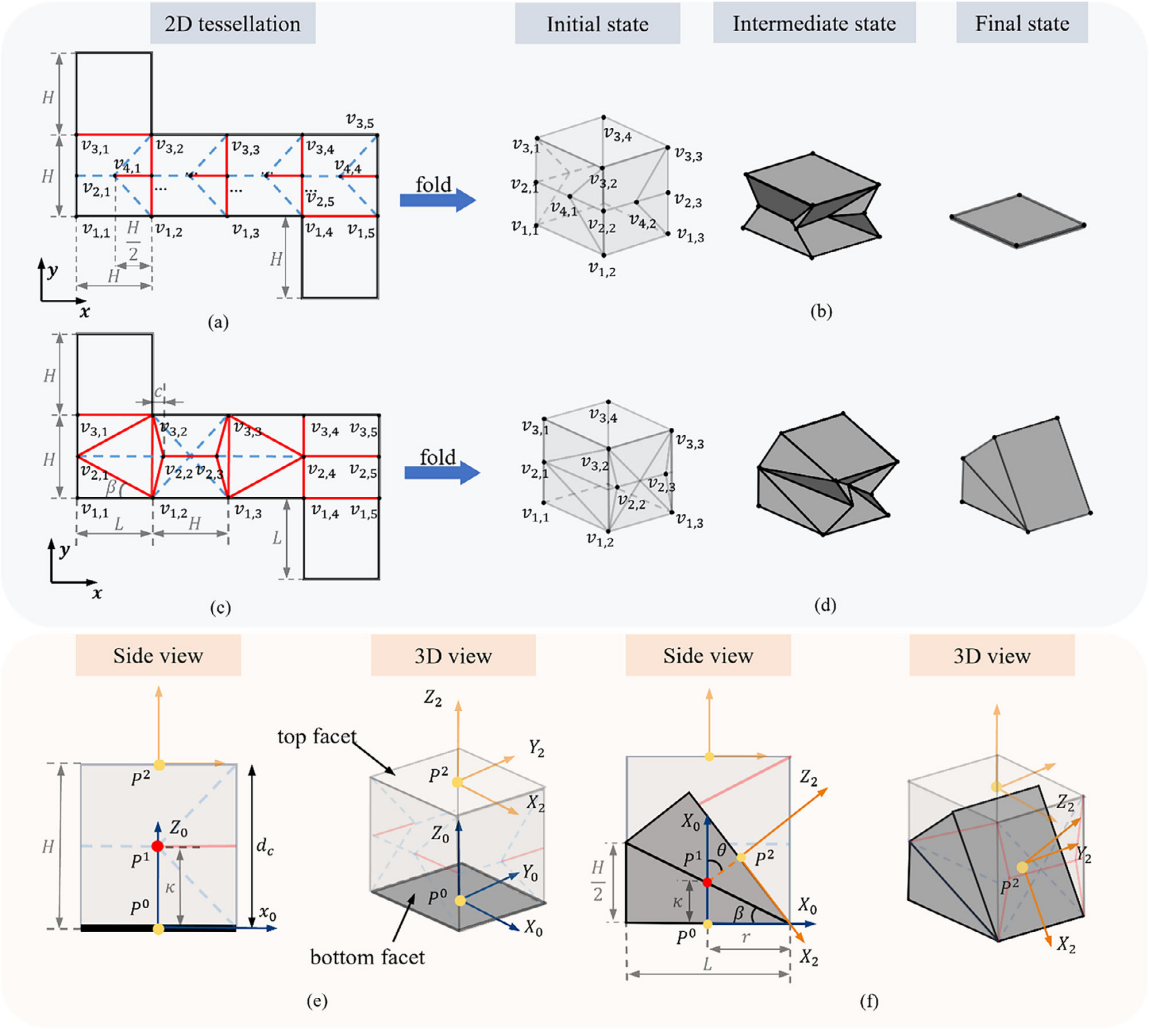
The 2D crease pattern, folding process, and geometry analysis of the origami actuators. a, b) illustrate the crease patterns and the folding process of the translational actuator, respectively, while c, d) show the corresponding details for the bending actuator. Blue dashed lines represent the valley folds, red solid lines are the mountain folds, and black lines are the paper edges. e, f) presents the relationship between the geometry and the motion range of the origami actuators without the influence of the material thickness. The yellow points represent the directional reference points, while the red points represent the positional reference points.

In this work, we analyze the geometrical design parameters of both origami actuators based on the zero‐thickness model to simplify the design process and accommodate the material thickness in the kinematics model. For the translational module, **Figure** **1**a,b illustrates the folding process to transform the 2D tessellation to a 3D actuator. As depicted in Figure 1a, the width of the actuator is set to be equal to the height for the simplification of the design process, and the geometrical parameter *H* is used to describe the translational module. The motion range of this actuator, as shown in Figure 1e, is characterized by the maximal contraction distance (*d_m_*), which in the zero‐thickness model equals to the module height, i.e., *d_m_* =  *H*. The bending actuator utilizes a waterbomb pattern on its collapsed facet to initiate bending movements,^[^ ^48^ ^]^ as illustrated in Figure 1c,d. The tessellation of this module is specified by two parameters: the height, *H*, and the length of the side facet, *L*. In the zero‐thickness module, the maximal bending angle θ_*m*_ can be expressed by θ_*m*_ =  2β, as shown in Figure 1f.

The 2D crease patterns (C) for the origami‐inspired actuators, delineated in Figure 1a,c, are defined by their respective geometrical configurations. These patterns are composed of vertices $V=\left\{\upsilon_{1,1},\upsilon_{1,2},\cdot \cdot \cdot ,\upsilon_{i,j},\cdot \cdot \cdot \right\}\in R^{2}$, where vertex ***v***_*i*,*j*_correspond to the coordinate on the *i*‐th row and *j*‐th column, denoted by the axes {***x***,  ***y***}. The crease patterns are further differentiated by their mountain folds, represented as red solid lines, and valley folds, indicated by blue dashed lines. Notably, an additional set of central vertices is incorporated within the translational pattern, as depicted in Figure 1a.

Upon folding up, these origami modules transform into LEGO‐like soft actuators with a single degree of freedom (DOF). To model their kinematics, as illustrated in Figure 1e,f, we define three reference points per actuator: ***P*** ^0^ and ***P*** ^2^ are defined as the directional reference points, located at the centers of the bottom and the top facets, and ***P*** ^1^ as the positional reference that marks the location of the actuator. For the translational actuator, ***P*** ^1^ is the midpoint of ***P*** ^0^ ***P*** ^2^; while for the bending actuator, ***P*** ^1^ is where extended lines from ***P*** ^0^ and ***P*** ^2^, perpendicular to their respective facets. Two local coordinate systems, {***X***_0_, ***Y***_0_, ***Z***_0_, ***P*** ^0^ } and {***X***_2_, ***Y***_2_, ***Z***_2_, ***P*** ^2^ } are then defined at the bottom and top facets, with the origins aligned with ***P*** ^0^ and ***P*** ^2^and ***Z*** axis perpendicular to the respective facets. The ***X*** axis oriented differs for the translational actuator; it is arbitrary due to axial symmetry, while for the bending actuator, it aligns with the bending direction. The ***Y*** axis is determined by the right‐hand rule. The kinematic model links these two local coordinate systems, aiding in the assembly strategy of complex robotic systems.

Crease patterns (C) for LEGO‐like origami actuators are determined by the motion range and size factor κ, which is the distance between ***P*** ^0^ and ***P*** ^1^ and is tailored to fit specific application scenarios. For the bending actuator, the maximal bending angle, θ_*m*_, and κ, are independent parameters. While for the translational actuator, the maximal translation is directly proportional to κ, with *d_m_* =  2κ. The independent variables dictating the folding pattern are collectively termed the crease parameter, P. For the translational actuator, P is solely defined by κ, whereas for the bending actuator, P is determined by θ_*m*_, and κ.

Once κ for the translational actuator, and κ and θ_*m*_ for the bending actuator are chosen, the crease parameter, P, and the forward kinematics model can be derived. Detailed computational methodology will be explicated in the Supporting Information.

#### 2.1.2 Kinematics Modeling with Thickness

**Figure 2:**
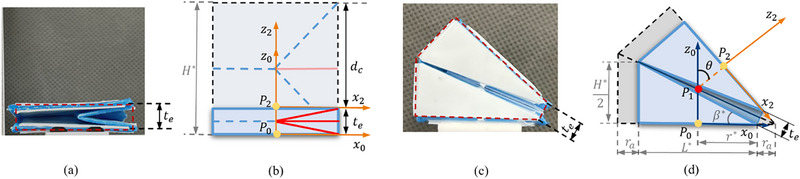
The thickness accommodation geometry analysis. a, c) show the practical actuated translational and bending origami actuator, and the thickness error is marked. b, d) illustrated how to decide the accommodated crease parameters ($P^{*}$).

In robotic systems, the physical prototyping of LEGO‐like origami actuators using engineering materials must consider material thickness, which significantly impacts the position accuracy of the origami actuators, as highlighted by previous research and our own experience.^[^ ^49^, ^50^ ^]^ As shown in **Figure** **2**a,c, the physical prototypes cannot be folded completely, where the red dashed line represents the limited folded state caused by the thickness. To address this issue, we propose a thickness accommodation model designed to reduce the position inaccuracy stemming from the disregard of material thickness. This model requires an adjustment to the crease parameter, P, which is the height, *H*, for translational actuator and the key design parameters, *H*, *L*, and β, for the bending actuator. In Figure 2b,d, the modified crease parameters are denoted with an asterisk (^*^), representing the accommodated parameters. The thickness incorporated forward kinematics model for the translational and bending actuator can be expressed by:

(1) $$T_{trans}\left(d\right)=\left[\begin{array}{c} \begin{array}{cccc} 1 & 0 & 0 & 0\\ 0 & 1 & 0 & 0\\ 0 & 0 & 1 & H^{*}-d\\ 0 & 0 & 0 & 1 \end{array} \end{array}\right]$$

(2) $$T_{bend}\left(\theta \right)=\left[\begin{array}{c} \begin{array}{cccc} \cos\theta & 0 & \sin\theta & L^{*}\sin\left(\vartheta^{*}\right)-\frac{L^{*}}{2}\\ 0 & 1 & 0 & 0\\ -\sin\theta & 0 & \cos\theta & L^{*}\cos\left(\vartheta^{*}\right)+\frac{H^{*}}{2}\\ 0 & 0 & 0 & 1 \end{array} \end{array}\right]$$

where $\vartheta^{*}=\theta +arctan\left(\frac{L^{*}}{H^{*}}\right)$ and $L^{*}=\frac{\sqrt{{H^{*}}^{2}+{L^{*}}^{2}}}{2}$ are for the simplification of expression. The detailed derivations are presented in Supporting Information.

In some cases, we need some passive links to connect the origami actuators and assemble the robot systems. The length of the link can be calculated as:

(3) $$l_{link,\;i}=∥P_{i-1}^{2}-P_{i}^{0}∥$$

Therefore, the transformation matrix of the link can be expressed as:

(4)

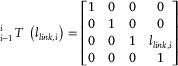




### 2.2 Optimization‐Based Assembly Map

Despite the modular nature of LEGO‐like origami soft actuators holding great promise for the swift construction of complex soft robotic systems, the rational assembly of these actuators remains a formidable challenge. This challenge arises from the requirement to seamlessly integrate various motion capabilities into a unified system that operates cohesively. Drawing parallels with rigid robots, the kinematics of origami soft actuators provides a useful tool, as individual motion determines the overall movement of a composite soft robotic system. In this section, we present an optimization‐based assembly strategy for such robotic systems. This strategy is aimed at minimizing the distance between the base and endpoint, assuring that the unique movement capabilities of each LEGO‐like soft actuator are satisfied, and avoiding potential obstacles.

**Figure 3:**
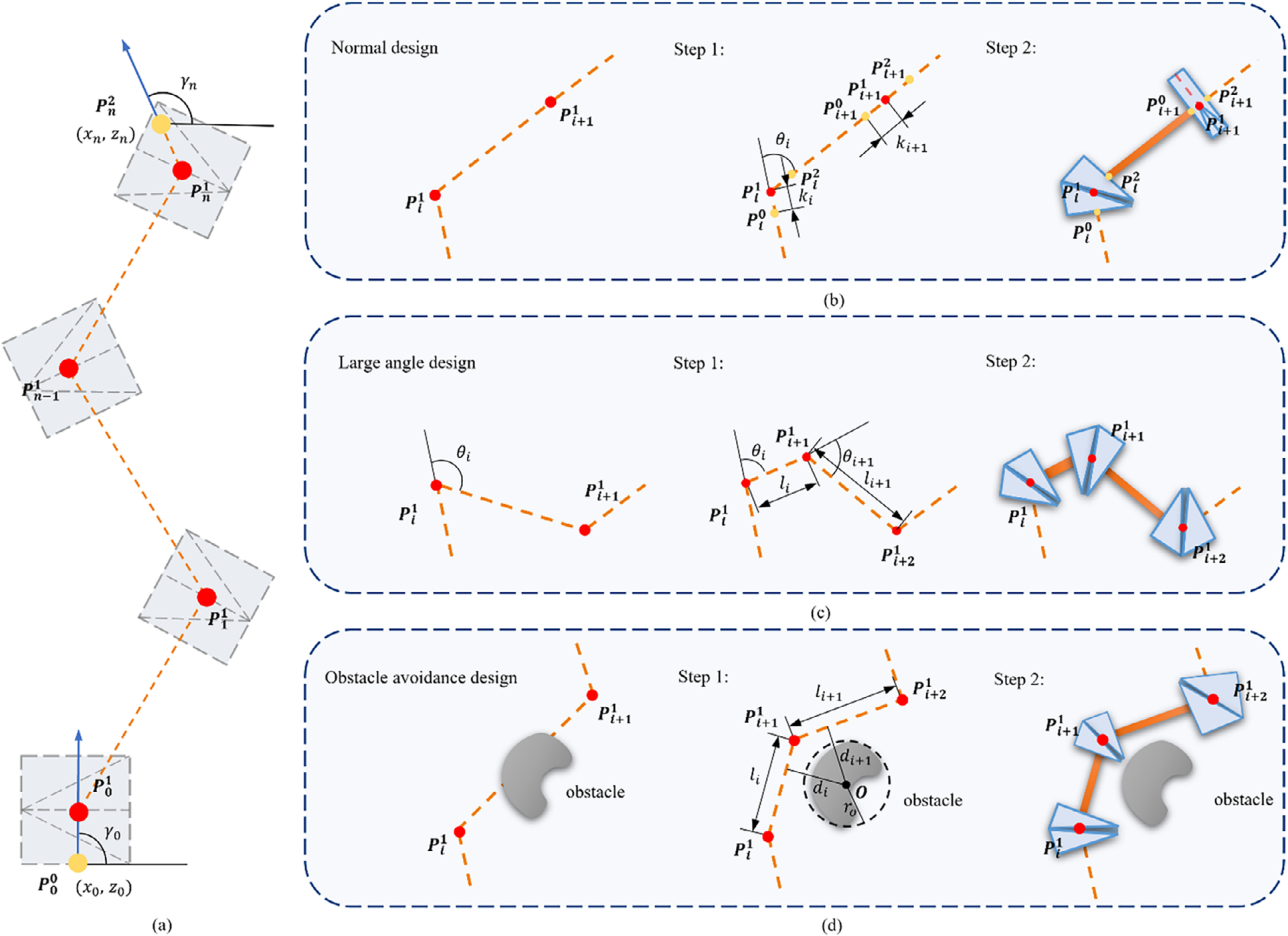
The design procedure of the LEGO‐like origami soft robotic system. a) shows the intended configuration of the LEGO‐like origami soft robotic system. b–d) illustrate the design procedure of free design, large‐angle design, and obstacle avoidance design, respectively. The red points represent positional reference points, while the yellow points represent directional reference points.

We define the origami actuator robotic system as a system that consists of (*n* + 1) LEGO‐like origami actuators connected in series with *n* passive links. Upon actuated, the origami actuators induce the robotic system to adopt a designated configuration through contraction and bending motions, as illustrated in **Figure** **3**. Within this framework, we define $P_{i}^{j}$ as the *j*‐th reference point of the *i*‐th actuator. The position and orientation of the starting point, $P_{0}^{0}$, and ending point, $P_{n}^{2}$, of the robotic system are predetermined by the intended function of the system. The design for an origami actuator robotic system subscribes to a two‐step optimization process.

#### 2.2.1 Step1: Reference Points Determination

The initial step involves delineating positional reference points, $P_{i}^{1}$, on the LEGO‐like origami actuators to facilitate seamless integration into the robotic system. The positional reference points of the first actuator and the endpoint, the *n*‐th actuator, are computationally determined by considering the orientations, γ_0_ and γ_*n*_, of the initial and terminal points, $P_{0}^{0}={\left[\begin{array}{ccc} x_{0} & y_{0} & z_{0} \end{array}\right]}^{T}$ and $P_{n}^{2}={\left[\begin{array}{ccc} x_{n} & y_{n} & z_{n} \end{array}\right]}^{T}$, in relation to *x*‐axis in the *xz* plane, as denoted in Figure 3a. The coordinates can be calculated by:

(5) $$\left\{\begin{array}{c} \;P_{0}^{1}={\left[\begin{array}{ccc} x_{0}+\kappa_{0}\cos\gamma_{0} & y_{0} & z_{0}+\kappa_{0}\sin\gamma_{0} \end{array}\right]}^{T}\;\\ \;P_{n}^{1}={\left[\begin{array}{ccc} x_{n}-\kappa_{n}\cos\gamma_{n} & y_{n} & z_{n}-\kappa_{n}\sin\gamma_{n} \end{array}\right]}^{T}\; \end{array}\right.$$

The positioning of $P_{i}^{1}$ for intermediate actuators is optimized by minimizing the length of linkages while ensuring operation within their designated ranges of motion, as illustrated in Figure 3b. This optimization challenge is mathematically articulated as follows:

(6) $$\min\sum_{i}^{n-1}∥P_{i}^{1}P_{i+1}^{1}∥\;s.t.\;\theta_{i}<\theta_{c}$$

with θ_*c*_ is the critical bending angle for the bending actuators.

In instances where the desired bending angle, θ, surpasses θ_*c*_ – tentatively set at θ_*c*_ = 90^○^ based on empirical evidence – a propensity for disproportionate height‐to‐length ratios may compromise stability. Our solution entails the introduction of an additional bending actuator to partition the bending angle, thereby facilitating the intended motion, as shown in Figure 3c. To determine the placement of this additional origami actuator, a cost function is proposed:

(7) $$\left\{\begin{array}{c} \;f_{a}=\sum_{i}^{i+1}\left(\lambda_{\Theta }\Theta_{i}+\frac{\lambda_{L}}{L_{i}}\right)\;\\ \;\Theta_{i}={\left|\frac{\theta_{i}}{\theta_{c}}\right|}^{m_{\Theta }}\;\\ \;L_{i}={\left(\frac{l_{i}}{∥P_{i+2}^{1}-P_{i}^{1}∥}\right)}^{m_{L}}\; \end{array}\right.$$

here, Θ_*i*_ signifies the relative bending angle of the *i*‐th bending actuator, with an ideal smaller Θ_*i*_ ensuring θ_*i*_ remains a safer range; $l_{i}=∥P_{i}^{1}-P_{i+1}^{1}∥$ denotes the distance between consecutive reference points; and *L_i_* indicates the relative length that is kept substantial to overlay close positioning of adjacent actuators. Adjustable weights λ_Θ_ and λ_*L*_, as well as exponents *m*_Θ_ and *m_L_*, are calibrated to balance the impact of bending angles and linkage lengths on the cost function. Typically, we set λ_Θ_ = 1, λ_*L*_ = 0.3, *m*_Θ_ = 5, and *m_L_* = 1. By minimizing the cost function *f_a_*, we ascertain the optimal position, $P_{i+1}^{1}$, for the additional origami actuator.

Analogs to the large bending angle scenario, the presence of an obstacle necessitates an extra bending actuator to circumvent it, as shown in Figure 3d. When positioning $P_{i+1}^{1}$, we model the obstacle as a circle centered at point ***O*** with radius *r_o_*, aiming to preserve the origami robot's configuration space whilst keeping a safe distance from the obstacle. We introduce an obstacle avoidance cost function, *f_o_*, to evaluate the placement of the new reference point:

(8) $$\left\{\begin{array}{c} \;f_{o}=\sum_{i}^{i+1}\left(\lambda_{D}D_{i}+\lambda_{L}L_{i}\right)\;\\ \;D_{i}={\left(\frac{r_{o}}{d_{i}}\right)}^{m_{D}}\; \end{array}\right.$$

where *d_i_* measures the distance between the center of the obstacle ***O*** and the segment $P_{i}^{1}P_{i+1}^{1}$; *D_i_* quantifies *d_i_* with reference to the obstacle's size, with a smaller *D_i_* indicating a safer configuration, and $L_{i}=∥P_{i}^{1}-P_{i+1}^{1}∥$ assesses the relative length, favoring a compact configuration that conserves materials. By modulating the weights λ_*D*_ and λ_*L*_, as well as the exponent *m_D_*, we balance safety with conservation of space. Typically, we opt for λ_*D*_ = λ_*L*_  = 1, and *m_D_* = 10. This approach facilitates the determination of the optimal location for $P_{i+1}^{1}$. Subsequently, we can specify the desired bending angles and size factors for the origami actuators using the aforementioned design methodology.

#### 2.2.2 Step2: Crease Parameter Calculation

In the second phase of the design process, we concentrate on the computation of crease parameters that are informed by the kinematics of the LEGO‐like origami actuators, ensuring effective functionality. Once the optimal positional reference points, $P_{i}^{1}$, of the LEGO‐like origami actuators have been identified, we select a proper size factor κ_*i*_ for each actuator. This enables the determination of the directional reference points, $P_{i}^{0}$ and $P_{i}^{2}$, positioned on either side of $P_{i}^{1}$, as shown in Figure 3a. Based on the directional reference points, we proceed to calculate the crease pattern parameters, $P^{*}$ and $C^{*}$ for the actuators using the procedures outlined in Algorithms 1–6, as detailed in Section 2.1 and Supporting Information. Subsequently, the kinematics of the actuators can be represented by Equations (1), (2), and (4).

The proposed methodology advocates for a systematic approach designed to devise and refine soft robotic systems equipped with LEGO‐like origami actuators. This methodological approach underpins the advancement of sophisticated soft robotic systems characterized by their enhanced versatility and operational capacity. The feasibility of the optimization‐based assembly strategy has been verified by Section 2.4.1 “System Position Accuracy Evaluation,” and Section 2.4.2 “Obstacle Avoidance Robot Arm” in Section 2.

### 2.3 Characterization of LEGO‐Like Origami Actuators

**Figure 4:**
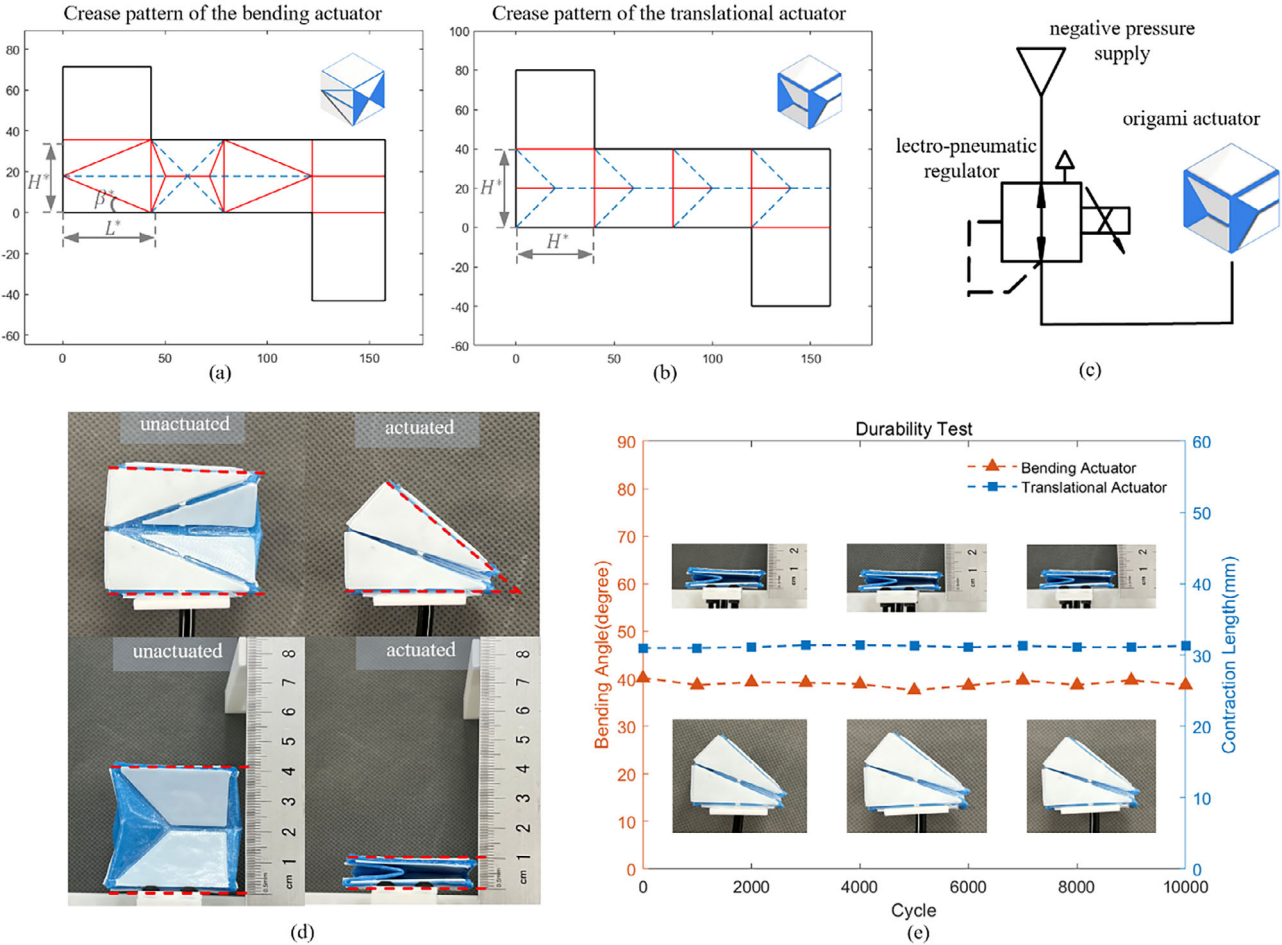
The experimental setup for position accuracy and durability tests, and the experiment results. a, b) show the thickness accommodated crease pattern of the characterized bending and translational actuators, respectively. c) shows the pneumatic circuit of the experimental setup. d) illustrates the free travel durability test of the bending actuator and translational actuator. e) is the durability repetitive test results of the origami actuators.

The LEGO‐like origami actuators are designed for high position accuracy and durability, with payload capacity as a vital attribute to their practical deployment. To assess their mechanical performance, we prototyped two actuators whose desired maximal range of motion is *d_m_* =  30 mm and θ_*m*_ = 40^○^ for the translational actuator and bending actuator, respectively. With size factor κ  = 15 mm and 10 mm, using Algorithms 1 and 3 in the Supporting Information, the key P for the translational actuator and bending actuator were calculated as *H_trans_* = 30 mm and {*L*,  *H_bend_*, β} = {54.9 mm, 40 mm, 20^○^}. After that, to reduce the thickness error, we recalculated the $P^{*}$ for both the actuators using Algorithms 5 and 6 in the Supporting Information. The revised parameters are $H_{trans}^{*}=$ 40 mm and $\left\{L^{*},\;H_{bend}^{*},\;\beta^{*}\right\}$ = {43.3 mm, 35.7 mm, 22.4^○^}, and the $C^{*}$ of both actuators are shown in **Figure** **4**a,b. After the fabrication, we established repeatable tests as shown in Figure 4c. The pneumatic setup is comprised of a vacuum pump capable of generating −80 kPa pressure with a flow rate of 15 L min^−1^ and an electro‐pneumatic regulator (SMC ITV2090‐212CL5) that modulates the pressure to −50 kPa.

#### 2.3.1 Position Accuracy and Durability Tests

The robustness of the origami actuators was gauged over 10 000 cycles of unrestrained operation. At every 1000 cycle‐juncture, we recorded the current position of the actuator: dihedral angles for the bending actuator and contraction for the translational actuator, as marked by the red dashed lines in Figure 4d. The experimental results presented in Figure 4e reveal that, through 10 000 cycles, the generated angle of the bending actuator remained consistently around 39.4° with a ±2.5% variance, resulting in an error of ≈0.6° compared to the desired bending angle of 40°. Similarly, the average contraction distance of the translational actuator was close to 31 mm, exceeding the desired contraction distance of 30 mm by 1 mm, with a variation of only ±1%. This consistency with minimal deviation signifies that the actuators possess outstanding durability, withstanding numerous cycles without suffering from fatigue or failure.

#### 2.3.2 Loading Tests

**Figure 5:**
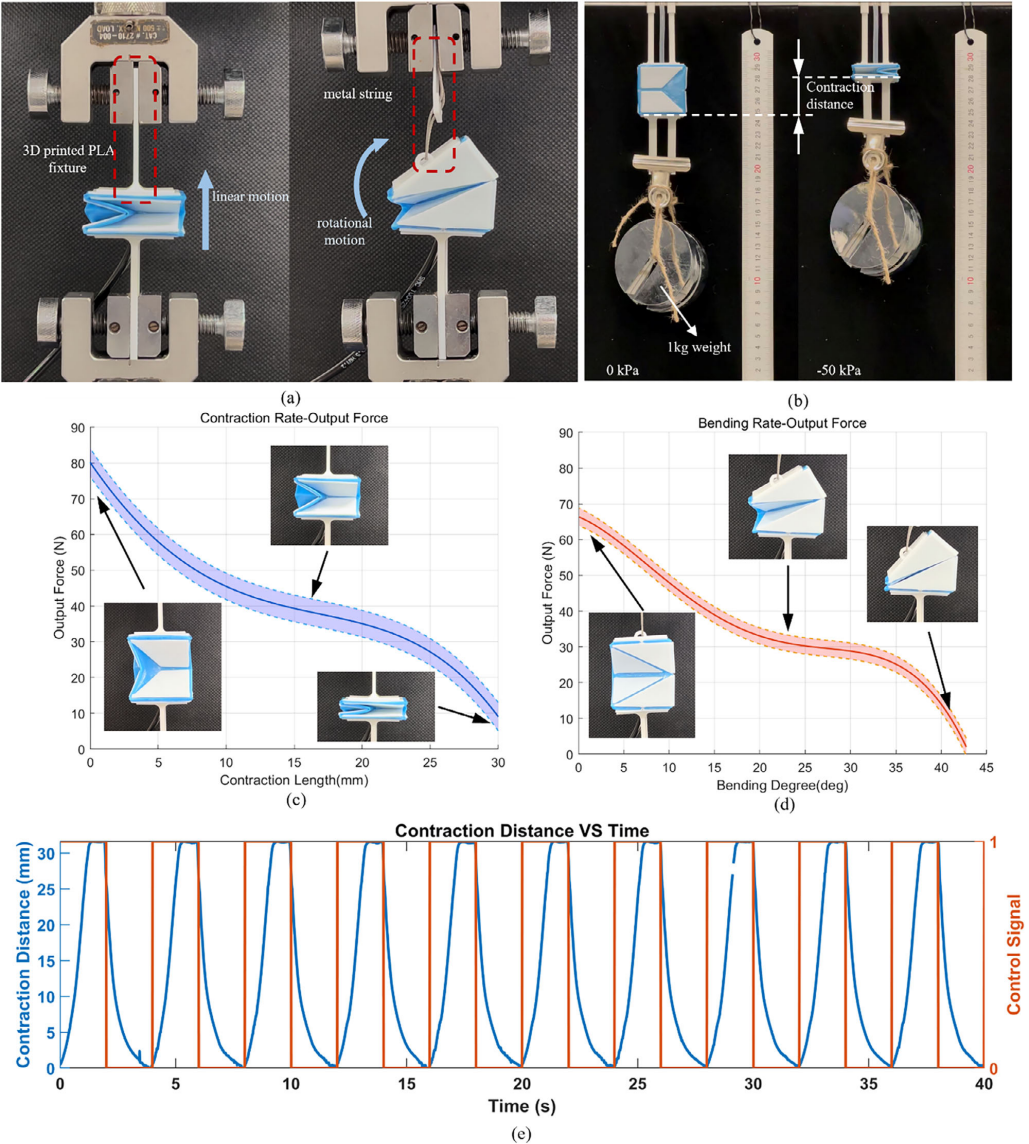
The loading test setup and experiment results. a) shows the blocked force tests of the translational and bending actuator, respectively. b) illustrates the setup for a high‐load test. c, d) are the blocked force test results of the translational and bending actuator, respectively. The solid lines indicate the average blocked force, while the shaded areas represent the error band. e) shows the contraction distance response corresponding to the repetitive high‐load test illustrated in (b).

The load‐bearing capabilities of the actuators were qualified by measuring the blocked force at various folding rates under a −50 kPa pressure setting and high‐load actuation experiments, as shown in **Figure** **5**a. In the blocked force tests, the bases of the translational actuator were clamped onto the tensile testing machine (Instron series 5500), while a metal string connected the tensile testing machine and the actuator to allow rotational motion of the top base. Under −50 kPa actuation pressure, the tensile machine measured the force exerted by the actuators as they transitioned from fully folded to completely unfolded states. This test was repeated five times, with results presented in Figure 5c,d. The findings indicate that the translational and bending actuators can exert peak blocked forces of ≈80 and 67 N at 0 folding rate with excellent repeatability, thus affirming their ability to handle heavy objects. Regarding the high‐load actuation tests, we used the translational module as a representative example. The weight of 1 kg was attached to the actuator, as shown in Figure 5b. A cyclic loading test was then conducted by applying −50 kPa and releasing it every 2 s following the square‐wave input (orange line in Figure 5e), while the contraction distance was recorded (blue line in Figure 5e). The results indicate that the actuator maintains stable performance under a high payload, confirming its reliability.

### 2.4 Demonstrations on LEGO‐Like Soft Robots

In this section, we assemble the LEGO‐like soft actuators into robotic arms and a walking robot to demonstrate the feasibility of the proposed standardized design paradigm.

#### 2.4.1 System Position Accuracy Evaluation

**Figure 6:**
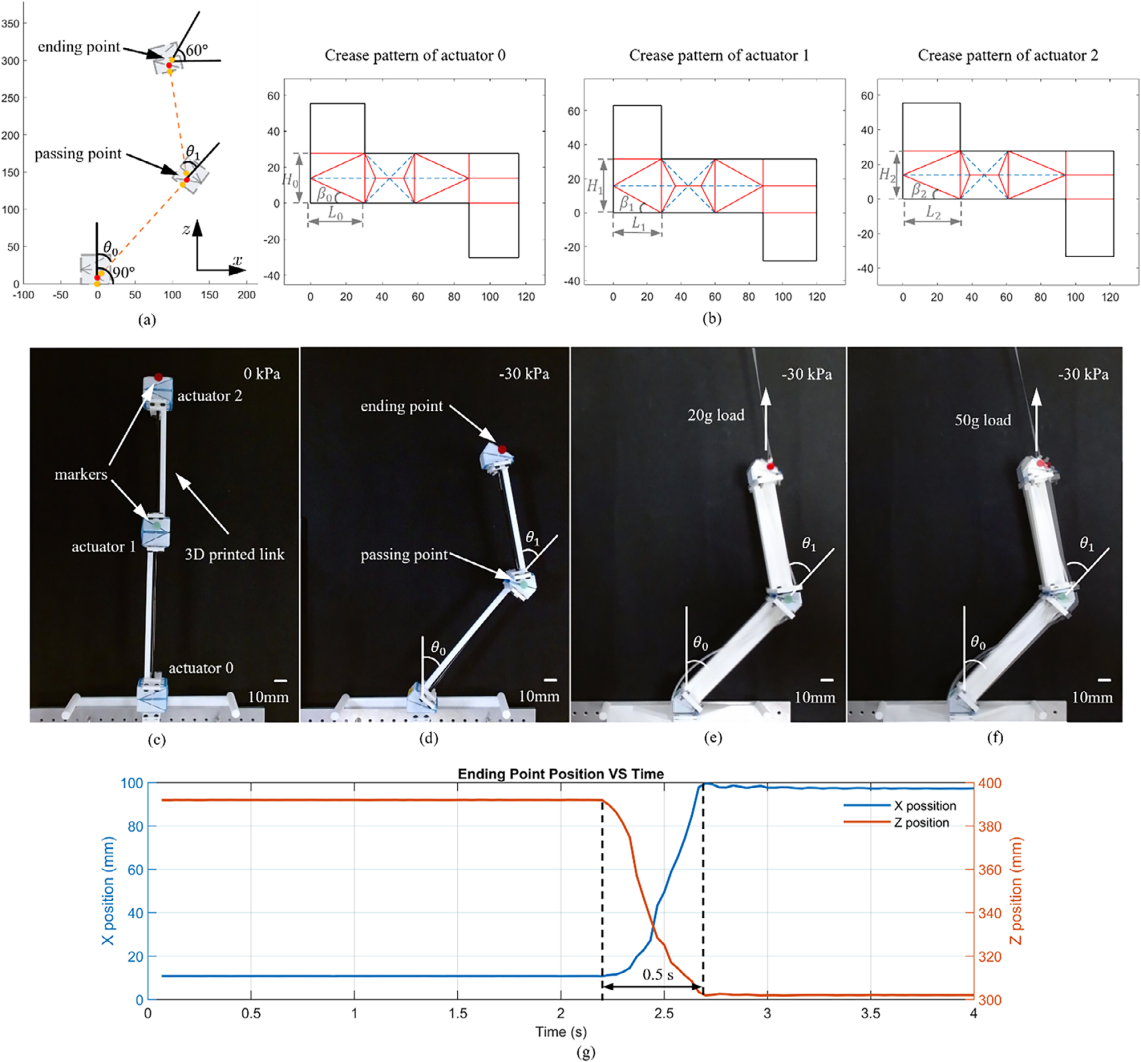
From the design to the real prototype of the origami actuator robotic system. a) illustrates the desired configuration of the robot arm and the reference points selection. b) depicts the crease patterns for the three origami actuators. c, d) show the assembled origami actuator robotic system and the activated configuration under free actuation, respectively. e, f) illustrate the changes in position accuracy when a load of 20 and 50 g was applied, respectively. g) shows the response time of the robotic system under the condition in (d).

The robotic arm, designed to operate within the *xz* plane as depicted in **Figure** **6**a, starts at the initial point $P_{0}^{0}$ with coordinates ${\left[\begin{array}{ccc} 0 & 0 & 0 \end{array}\right]}^{T}$ and an orientation angle of γ_0_ =  90^○^. The endpoint coincides with $P_{2}^{2}$ at ${\left[\begin{array}{ccc} 100 & 0 & 300 \end{array}\right]}^{T}$ with orientation of γ_*n*_ =  60^○^. Positioned between these two points is the passing point $P_{1}^{1}$ at ${\left[\begin{array}{ccc} 120 & 0 & 140 \end{array}\right]}^{T}$. To ensure proper sizing for all actuators, we assigned κ_0_  = κ_2_ = 8 mm and κ_1_  = 9 mm. Calculation using Equation (5) determined the positional reference point $P_{0}^{1}={\left[\begin{array}{ccc} 0 & 0 & 8 \end{array}\right]}^{T}$ and $P_{2}^{1}$ = ${\left[\begin{array}{ccc} 96 & 0 & 293 \end{array}\right]}^{T}$. The desired bending angles for the actuators were computed to be θ_0_ = 42.3^○^, θ_1_ = 51.2^○^, and θ_2_ = 38.9^○^. The length of the two links were *l*_*link*, 1_ =  161.4 mm and *l*_*link*, 2_ =  137.9 mm according to the calculation. Utilizing the bending angles and size factors, the crease parameters for the actuators were calculated based on the model described in Section 2.1. Table S3 (Supporting Information) lists the computed normal crease parameters P and thickness‐accommodated $P^{*}$ for all actuators, revealing significant deviations that underscore the importance of the thickness accommodation. The corresponding $C^{*}$ for the actuators are shown in Figure 6b. The $P^{*}$ were used to design and fabricate origami actuators, which were then assemble with 3D printed PLA links as shown in Figure 6c.

Prior to performing physical experiments, the proposed assembly strategy was validated through a forward kinematics model denoted in Section 2.1. The ending position was calculated by substituting the geometrical parameters into Equations (2) and (4), and was given by:

(9)

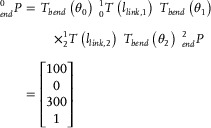

herein, 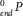 is the position of the endpoint in the global coordinate frame, and 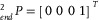 is the position in the local coordinate system. It can be concluded that the proposed assembly can achieve the desired configuration, as the computed endpoint matches the designated endpoint.

For position accuracy characterizations, we conducted 10 repeatable tests on the assembly robotic arm equipped with color‐marked origami actuators for reference. Under a −30 kPa vacuum pressure, the linkage system achieved the desired configuration, captured by a camera and depicted in Figure 6d. The positional and angular errors of the reference points were analyzed over the 10 loading cycles, as shown in **Table** **1**. The position error is 3 mm, and the angular errors of actuator 0 and 1 are −1.27^○^ and 0.95^○^,^[^ ^51^ ^]^ respectively, confirming high position accuracy and reliability of the origami robotic system. The time‐position response of the ending point is shown in Figure 6g. The entire process was completed within 0.5 s, demonstrating the rapid response of the LEGO‐like origami actuator robotic system.

**Table 1:** The Comparison of the Desired Position and Experimental Data under No Load.

	Position	Average	Design	Position error
Ending point	x position (mm)	97.92	100	−2.08
	z position (mm)	302.19	300	2.19
Passing point	x position (mm)	120.32	120	0.32
	z position (mm)	144.91	140	4.91
Actuator 0	θ_0_ (^○^)	41.03	42.3	−1.27 (3%)
Actuator 1	θ_1_ (^○^)	52.15	51.2	0.95 (1.9%)

Furthermore, we conducted experiments with 20 and 50 g weights to verify the position accuracy under varying loads, as shown in Figure 6e,f. To minimize the influence of the 3D‐printed rigid links, we reprinted the links with increased width while keeping the original length design. The results, summarized in **Table** **2**, show that the robotic system maintained relatively high accuracy, with deviations within 1 and 7 mm under 20 and 50 g loads, respectively. These findings demonstrate the system's potential to perform tasks effectively under such loading conditions.

**Table 2:** The Comparison of Position Accuracy under Varying Loads.

	Position	Design	No load	20g load	50g load
Ending point	x position (mm)	100	102.9	102.6	97.4
	z position (mm)	300	298.7	298.9	302.2
Passing point	x position (mm)	120	124.6	123.7	122.4
	z position (mm)	140	137.9	137.9	140.5
Actuator 0	θ_0_ (^○^)	42.3	42.2	41.9	41.0
Actuator 1	θ_1_ (^○^)	51.2	51.5	51.2	51.6

#### 2.4.2 Obstacle Avoidance Robot Arm

This experiment was designed to validate the effectiveness of the optimization‐based method for obstacle avoidance and large‐angle avoidance in the context of LEGO‐like origami actuators. A plate was placed at ${\left[\begin{array}{ccc} 180\; & 0 & 50\; \end{array}\right]}^{T}$ on the table, with the objective of the robot arm being to accurately place a toy hamburger onto this plate. An additional challenge was introduced by an obstacle situated at ${\left[\begin{array}{ccc} 80\; & 0 & 40\; \end{array}\right]}^{T}$, with a radius of 30 mm, located between the plate and the robot arm. It was thus imperative to design a robot arm capable of effectively navigating around this obstacle. The initial point for the robot arm was set at ${\left[\begin{array}{ccc} 0 & 0 & 0 \end{array}\right]}^{T}$ with an initial direction of 90^○^. Taking into account the size of the toy hamburger, the ending point was set at ${\left[\begin{array}{ccc} 150 & 0 & 50 \end{array}\right]}^{T}$ with an angle of 0^○^ to ensure its positioning above the plate after actuation.

**Figure 7:**
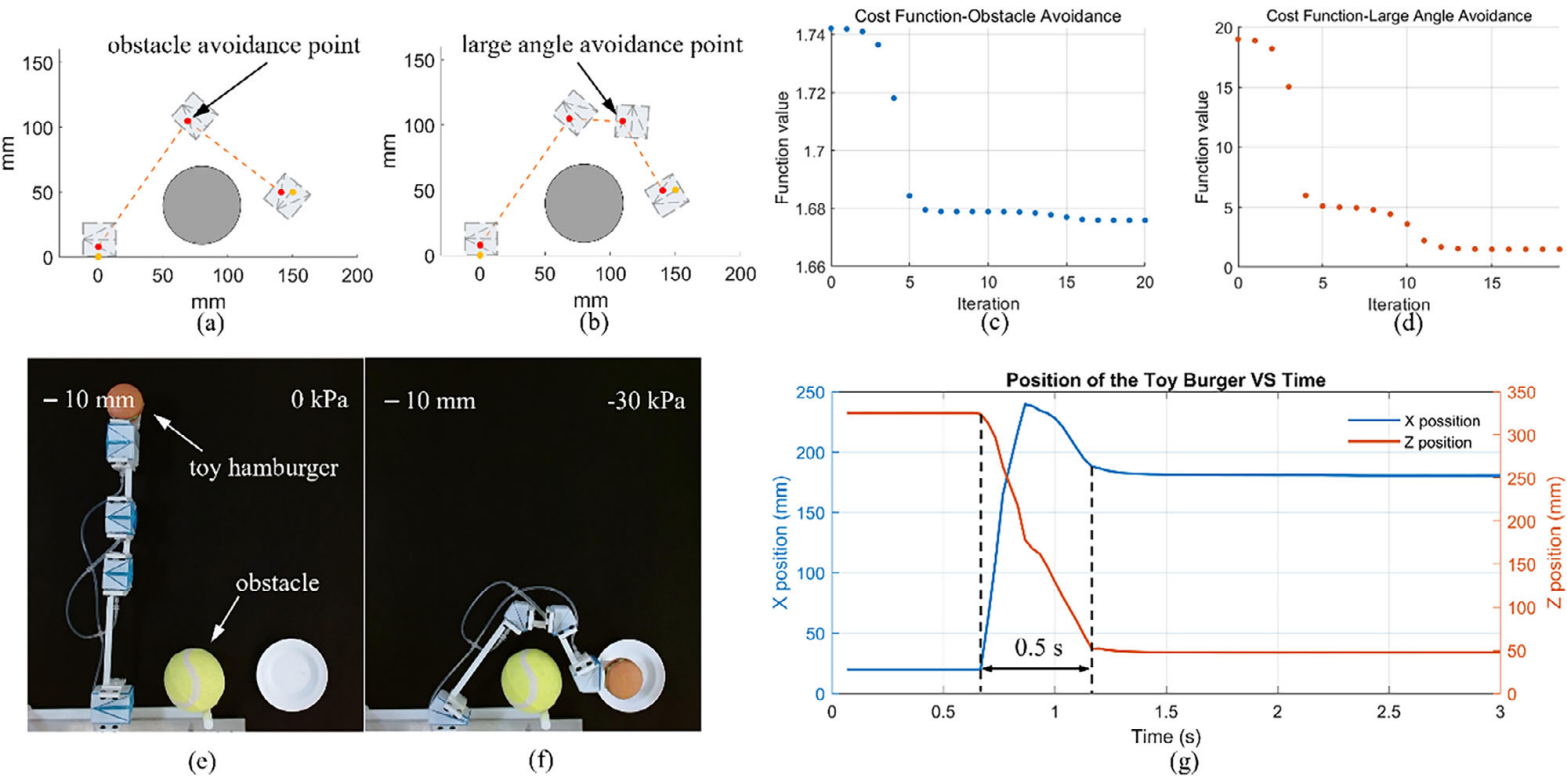
The design procedure, assembly, and actuation of the obstacle‐avoidance robot. a) shows the result of the obstacle avoidance design. An extra positional reference point is introduced, and c) is the corresponding iteration history of the cost function ***f***_***o***_. b) illustrates the result of implementing the large‐angle avoidance design, while d) is the iteration history of the cost function ***f***_***a***_. e) shows the assembled robot arm according to the optimization‐based design method, and f) is the configuration after providing a negative pressure of −30 kPa. g) illustrates the response of the robotics system.

As depicted in **Figure** **7**a, we set the size factors for the first and last LEGO‐like origami actuators as κ_0_ = 8 mm and κ_2_ = 10 mm, respectively. Through Equation (5), we ascertained $P_{0}^{1}={\left[\begin{array}{ccc} 0 & 0 & 8 \end{array}\right]}^{T}$ and $P_{2}^{1}={\left[\begin{array}{ccc} 140 & 0 & 50 \end{array}\right]}^{T}$. Using Equation (8), the intermediate point was iteratively found to be ${\left[\begin{array}{ccc} 68.8 & 0 & 105.0 \end{array}\right]}^{T}$, corresponding to the obstacle avoidance point $P_{1}^{1}$, as depicted in Figure 7a. The iteration history is shown in Figure 7c. Subsequently, the calculated bending angle for the additional origami actuator θ_1_ = 92.3^○^, was found to exceed the critical bending angle defined in Section 2.2. To prevent potential instability issues arising from the significant height‐to‐length ratio of the actuator, an additional origami actuator was incorporated to mitigate θ_1_. This step involved the sequential reallocation of the positional reference points for the LEGO‐like origami actuators. Figure 7b illustrates the recalculated position for the large‐angle avoidance point $P_{2}^{1}$ as ${\left[\begin{array}{ccc} 109.4 & 0 & 102.7 \end{array}\right]}^{T}$ using Equation (7), while the remaining reference points were kept unchanged. The optimization trajectories for the cost function *f_a_* is depicted in Figure 7d. Following the optimization, the desired bending angle for the four origami actuators were θ_0_ = 35.4^○^, θ_1_ = 57.9^○^, θ_2_ = 56.6^○^, and θ_3_ = −59.9^○^, each below the critical bending angle. Optimization for both obstacle avoidance and large angle avoidance was executed using the “fmincon” in MATLAB R2023b, with initial guesses set at ${\left[\begin{array}{ccc} 80 & 0 & 100 \end{array}\right]}^{T}$ and ${\left[\begin{array}{ccc} 200 & 0 & 80 \end{array}\right]}^{T}$, respectively.

Once the positional reference points were determined, Algorithms 3, 6, and 4 in the Supporting Information were employed to derive the thickness accommodated crease parameter $P^{*}$ for all actuators as {*L*_0_*,*H*_0_*, β_0_*} = {38.7 mm, 28.3 mm, 20.1^○^}, {*L*_1_*,*H*_1_*, β_1_*} = {25.3 mm, 32.0 mm, 32.3^○^}, {*L*_2_*,*H*_2_*, β_2_*} = {26.0 mm, 32.0 mm, 31.6^○^}, and {*L*_3_*,*H*_3_*, β_3_*} = {27.7 mm, 36.0 mm, 33.0^○^}. Equation (4) enabled the calculation of the link lengths as *l*_*link*, 1_ =  101.9 mm, *l*_*link*, 2_ =  22.7 mm, and *l*_*link*, 3_ =  41.9 mm. Following fabrication of the actuators using lamination techniques and the links through 3D printing, the assembly was completed as shown in Figure 7e. When a negative pressure of ‐30 kPa was applied, the robot arm successfully maneuvered around the obstacle and deposited the toy hamburger onto the plate, as illustrated in Figure 7f and Movie S1 (Supporting Information). The time‐position response of the attached toy is shown in Figure 7g. The final position stabilized to ${\left[\begin{array}{ccc} 180.5\; & 0 & 47.3\; \end{array}\right]}^{T}$ within 0.5 s, demonstrating the rapid response of the LEGO‐like origami actuator robotic system. This experiment confirms the reliability of the proposed assembly strategy for obstacle and large‐angle avoidance in LEGO‐like origami actuator robot arm systems.

#### 2.4.3 Tight Space Inspection Robot

**Figure 8:**
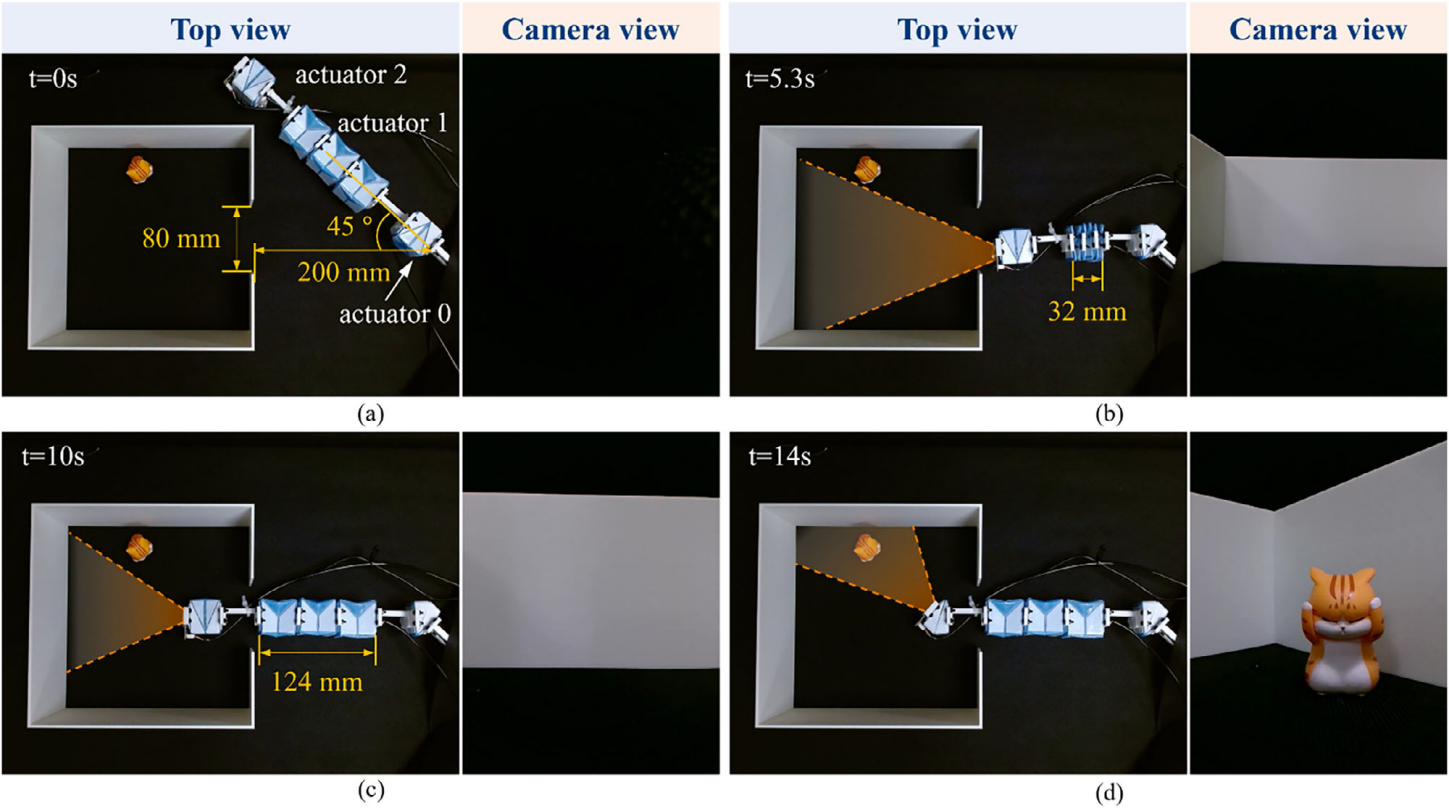
Demonstration of a tight space inspection robot arm with a micro camera. The orange shaded area is the image‐capturing range. a) shows the initial state, no actuator is activated. In (b), the translational actuators first contract to 32 mm, and the bending actuator rotates an angle of 45^○^. c) show the robot arm going through the opening by releasing the translational actuators. d) illustrates the actuator 2 rotates an angle of 45^○^, and the micro‐camera captures the toy cat inside the box.

The robotics arms previously used for position accuracy characterization were controlled by a single negative pressure supply. It is crucial to note that these actuators can also function independently to accommodate more complex applications. In this section, we have integrated translational and bending actuators with various actuation sequences to conceive a robotic system proficient in space inspection within constrained environments. As shown in **Figure** **8**a, a toy cat is positioned within a box measuring 250 mm on each side, with an 80 mm opening on one facet. The robot initiates 200 mm from the opening at a 45° orientation toward it. The challenge is to design a robot capable of maneuvering through the opening to locate the cat.

The robot consists of two bending actuators, three combined translational actuators, and two linkage sections. For ease of description, we sequentially labeled the origami actuators from 0 to 2, as depicted in Figure 8a. Actuators 0 and 2 were designed to achieve a 45° bending angle, with crease parameters {L*, H*,  β*} = {39.1 mm, 36.2 mm, 24.9^○^} as determined by the proposed optimization‐based design methodology. The translational actuators were capable of adjusting their length between 10 and 40 mm in activated and inactivated states, with H* = 40 mm as calculated using our design algorithm. A micro‐camera attached to the end of actuator 2 captures the robot's viewpoint. Initially, all origami actuators were exposed to atmospheric pressure (Figure 8a). Negative pressure of −30 kPa was then applied to actuator 1, followed by pressure supplied to actuator 0, prompting the robot to pivot 45^○^, as depicted in Figure 8b. Upon releasing actuator 1, it reverted to its original length, allowing the to pass through the box opening (Figure 5c). Finally, activating actuator 2 caused the camera to rotate 45^○^, capturing the toy cat inside the box, as shown in Figure 8d. Movie S2 (Supporting Information) showcases the overall action of the tight space inspection robot. This task demonstrates the capabilities of our LEGO‐like origami actuators in controlling the robot through various configurations, highlighting their potential utility in constructing reconfigurable robotic systems.

#### 2.4.4 Bipedal Walking Robot

**Figure 9:**
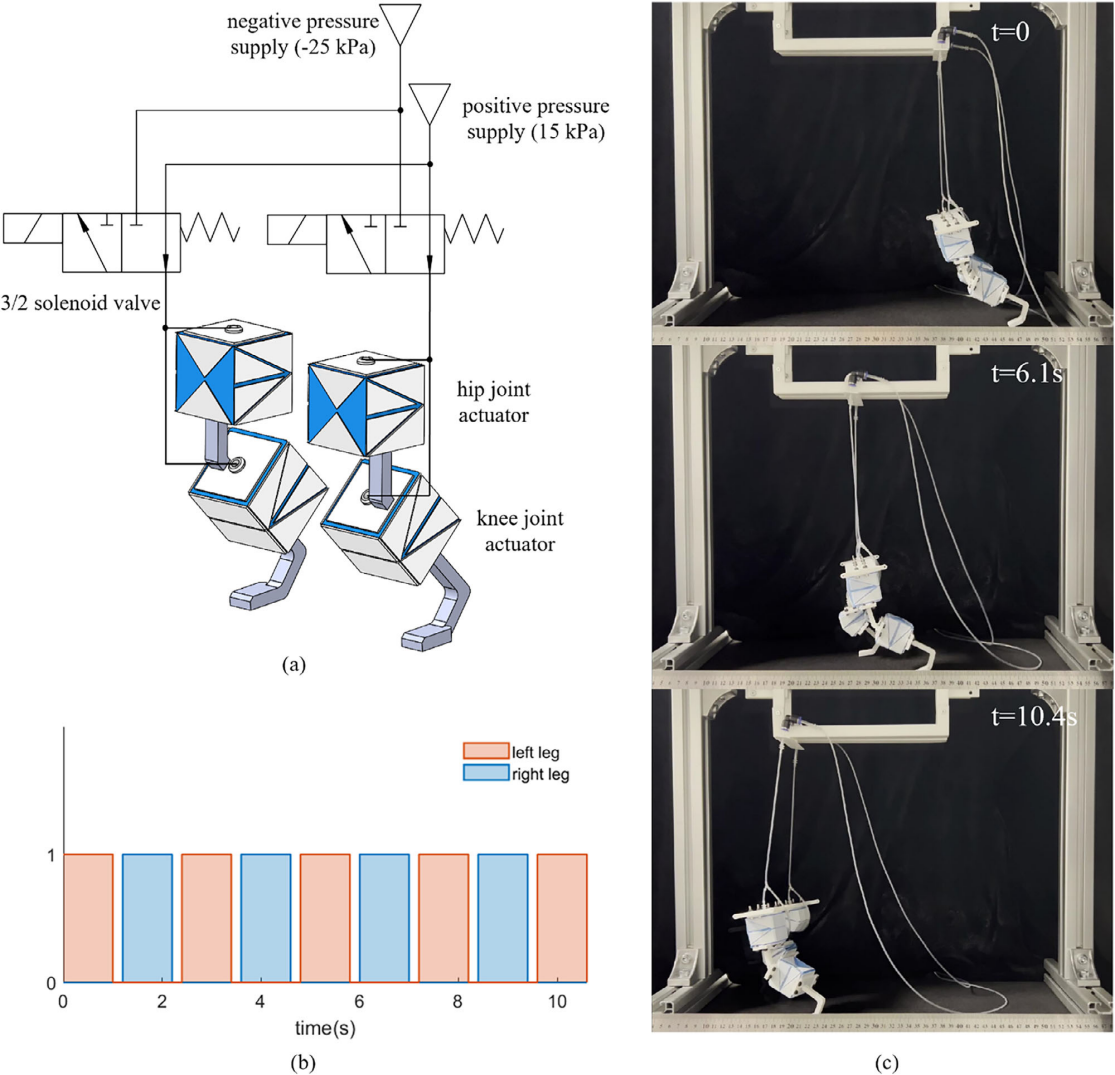
The actuation pneumatic circuit, CAD model of the bipedal robot, and the forward motion of the robot. a) shows the robot is connected to two pressure supplies. b) shows the control signal sequence. c) illustrates the bipedal robot walking around 24 mm in 10.4 s.

To further exhibit the versatility of the proposed origami actuators, we built a bipedal robot utilizing origami actuators as joints. In this task, the hip and knee joints are expected to achieve a bending angle of 45°. As presented in **Figure** **9**a, each leg of the robot integrates two actuators described in Section 2.4.2, with crease parameters {L*, H*,  β*} = {39.1 mm, 36.2 mm, 24.9^○^}, representing the hip and the knee joints. In addition to a negative pressure source, a positive pressure source of 15 kPa was also employed to ensure rapid reversion of the actuators. These pressure sources were interfaced with the pressure and exhaust ports of the actuators, with both hip and knee joints on the same leg connected to the output port of the solenoid valve. The actuators were normally subjected to positive pressure but could be switched to negative pressure by a 3/2 solenoid valve upon receiving a 6 V electro signal.

The actuation sequence is shown in Figure 9b. A “1” indicates a high voltage signal (6 V), whereas a “0” signifies a low voltage signal (0 V). The legs were activated alternatively by supplying a high voltage signal to each leg for 1 s, followed by a low voltage for 0.2 s. Notably, the air tubing also served as a stabilizing feature, aiding in maintaining balance and reducing the risk of the robot falling during its movement. Figure 9c illustrates the forward walking motion of the robot, traversing ≈240 mm within 10.4 s, equating to a speed of ≈23.1 mm s^−1^. The overall action of the bipedal robot is shown in Movie S3 (Supporting Information).

## 3 Conclusion and Discussion

The soft robotics society confronts two primary challenges that have to date limited its full‐scale adoption: the high cost associated with designing complex systems and the limitations in position accuracy of traditional stretch‐dominated soft actuators. Our pioneering work in LEGO‐like origami soft actuators sets forth a path to overcome the prevalent challenges in soft robotics. In this work, we have proposed LEGO‐like modular soft actuators and developed an innovative optimization‐based design methodology that standardizes the design of soft robotic systems. These actuators are ingeniously crafted to account for material thickness, affording them high‐accuracy bending and contraction capabilities due to their fold‐driven kinematics. The methodology enables the creation of an origami robotic system capable of adopting configurations that are either freely selected or tailored to navigate around obstacles. Once the desired configuration is determined, our approach can efficiently calculate the necessary crease parameters for each actuator and generate the corresponding crease patterns. For the fabrication process, we adopted a 3D printing facilitated lamination technique to guarantee high fidelity construction of the actuators. The 3D printed origami panels are bonded onto flexible TPU‐coated fabric through thermo‐compression, ensuring that deformations occur only at the designated hinge locations.

The characterization experiments performed on our origami actuators showcase remarkable capabilities. The translational actuator can handle loads up to 80 N at −50 kPa and operates stably under high‐load conditions. However, dynamic loading tests remain to be performed and will be addressed in future work. Both types of actuators have shown remarkable durability over 10000 cycles with minimal actuator performance. Subsequent experiments have validated the accuracy of our assembly strategy and the effectiveness of the thickness accommodation method. This was accomplished by precisely measuring the final posture of the prototype robotic arms, thus confirming the accuracy of our design techniques. To showcase the versatility of our origami actuators, we designed and tested a tight space inspection robot and a bipedal walking robot. Both application scenarios demonstrate the feasibility of our LEGO‐like origami actuators and optimization‐based design methods in practical scenarios.

The advent of LEGO‐like origami robotic systems is poised to significantly impact the field of soft robotics. Despite this progress, current origami patterns are limited to basic motions such as contraction and bending. It is imperative to develop new patterns that can fabricate actuators capable of executing more intricate movements. Furthermore, for each new task, a new actuator pattern may need to be designed, and our current system utilizes simple on‐off solenoid valves for actuator control. This could be a limitation for the existing framework. However, we would like to highlight again that all actuators in our system are reusable. Within acceptable error tolerances, previously fabricated actuators can still be repurposed to assemble different robotic systems. In scenarios where task environments are complex and changeable or single‐use applications, our LEGO‐like origami actuators offer a cost‐effective alternative to conventional robotics systems. In addition, it is worth noting that incorporating proportional valves could enable continuous control over the actuators to achieve any position within their motion range and further enhance their modularity.

In future research, the development of a closed‐loop controller for the proposed origami actuator system would further improve its accuracy and versatility. We plan to develop a pneumatic controller for large‐scale soft robotics systems. Integrating a large number of modular origami actuators (e.g., more than 10 or 20 modules in the robotic system) can enable more complex and versatile motions. Such highly modular and functionally rich soft robotic systems remain rare. In this work, the obstacle avoidance task is limited to a single‐obstacle scenario, where simultaneous activation of all actuators is sufficient to achieve avoidance. In more complex environments, however, coordinated actuation sequences become essential. At present, our obstacle avoidance model does not account for multi‐obstacle cases. Future efforts will focus on developing a more comprehensive strategy to address multi‐obstacle scenarios, which would further enhance the applicability and significance of this work.

In addition, the incorporation of a closed‐loop system would enable the inclusion of time control in the optimization algorithm, further enhancing the automation capabilities of the LEGO‐like origami actuator systems. A vision‐based system, such as a camera, presents a promising solution for this purpose, as it offers a lightweight alternative to traditional rotational encoders, which are generally unsuitable for flexible and lightweight soft robotic platforms. Additionally, in our future development, we intend to explore a wider array of origami patterns that extend beyond existing designs. This advancement will enhance the versatility and adaptability of the actuators, allowing them to undertake more complex and diverse tasks.

## 4 Experimental Section

### Fabrication Process and Material Choices

Origami‐inspired structures leverage folding techniques to yield complex 3D shapes. This fabrication method emphasized maintaining actuator facet rigidity while assuring deformation solely at flexible fabric materials. This was achieved by a dual‐material strategy, which involved using nondeformable 3D‐printed polylactic acid (PLA) for the rigid facets and flexible thermoplastic polyurethane‐coated nylon fabric (TPU‐coated fabric) for the hinges. The PLA facets were bonded to flexible TPU‐coated fabric through thermo‐compression. Additionally, the TPU‐coated fabric also ensured the airtightness necessary for pneumatic actuation. Accurate placement of individual facets onto the TPU‐coated fabric was secured through temporary positioning bridges between facets.^[^ ^46^, ^47^ ^]^

The fabrication process is illustrated in Figure S1 (Supporting Information) with four steps. First, the PLA panels were printed with a thickness of 1 mm utilizing a fused deposition modeling (FDM) 3D printer (Bambu Lab X1‐Carbon Combo). Panels that did not contribute significantly to the final folded structure, such as the small triangle panel ***v***_4,1_ ***v***_3,2_ ***v***_4,2_, were omitted to ensure a high folding ratio. Second, the 3D printed panels were tessellated onto the TPU‐coated fabric using a thermo‐compression machine, which was set at 175 °C for 8 s. An additional 0.05 mm pure TPU film was introduced between the PLA and the coated fabric to enhance bonding. Following this, the positioning bridges were cut off, folded them into a 3D actuator, and seal it through ultrasonic welding. Finally, an air vent was attached for integration with the pneumatic controller. When assembling different LEGO‐like actuators, they were connected using short air tubes and screws, as shown in Figure S2 (Supporting Information). The air tubes were inserted into the vents with an interference fit to enhance airtightness, while the screws provide sufficient mechanical strength for secure connection.

## Conflict of Interest

The authors declare no conflict of interest.

## Supporting information



Supporting Materials

Supplemental Movie 1

Supplemental Movie 2

Supplemental Movie 3

## Data Availability

The data that support the findings of this study are available from the corresponding author upon reasonable request.
