# Supplementary material for: LEGO‐like Origami Robots Standardize Structure Design of Soft Robots
Source: Adv Sci (Weinh). 2025 Dec 12;13(5):e13881. doi: 10.1002/advs.202513881 (PMC12849906; doi:10.1002/advs.202513881)
Supplement: Supplementary file 1 — Supporting Materials [file ADVS-13-e13881-s001.docx]

Supporting Information

LEGO-like Origami Robots Standardize Structure Design of Soft Robots

Zheng Wang, Yuzhe Wang, and Hongying Zhang*

Zheng Wang, Hongying Zhang

Department of Mechanical Engineering, National University of Singapore, Singapore, Singapore
E-mail: [hy.zhang@nus.edu.sg](mailto:hy.zhang@nus.edu.sg), Hongying Zhang

Yuzhe Wang

Singapore Institute of Manufacturing Technology, Agency for Science, Technology and Research, Singapore, Singapore

**This supporting information file includes:**

1. Review of Existing Origami Actuators
2. 2D Tessellation Generation
3. Kinematics Modelling with Thickness
4. Algorithm 1 to 6
5. Figure S1 to S2
6. Table S1 to S3
7. Legends for Movie S1 to S3
8. **Review of Existing Origami Actuators**

As shown in Table S1, we review the existing origami actuator designs based on five key aspects: modular design, automatic assembly, kinematics modeling with thickness, module position accuracy / unit size, and the capacity for rebuilding different robots. These aspects are essential for evaluating the standardization level and practical applicability of origami actuators.

The modular design concept contributes to standardization and simplifies the overall design process. Then, we reviewed if a standardized assembly map has been proposed, which is the core contribution to simplify the design process of soft robotics. Subsequently, we evaluate whether the error introduced by material thickness has been accounted for, and whether any compensation methods have been implemented during the design phase, as this factor significantly affects position accuracy,^[1,2]^ which is particularly important for evaluating the performance of a robotic system. To ensure a fair comparison, we focus on the accuracy of individual modules rather than entire systems. Lastly, we also reviewed if the proposed design has been used to develop different robotic systems, which can effectively prove the feasibility of proposed works.

Parallel folding, the simplest origami structure with folds aligned in parallel, is exemplified in Peano-HASEL and pouch motors.^[3,4]^ Both designs utilize thin materials and are not modularized, and reports on their position accuracy are rare. The Kresling pattern, formed by compressing thin-walled cylinders with distortion on the two ends, has been used by A. E. Forte et al., C. Zhang et al. and T. Jin et al. to design bistable origami modules.^[5-7]^ Although these designs have demonstrated potential for assembling various robotic systems, these origami actuators lack position accuracy data. Inspired by the waterbomb pattern, C. H. Belke et al. designed Mori,^[8]^ which offers reconfigurable structures but lacks position accuracy for one module. J. Santoso et al. and Z. Zhang et al. created cable-driven origami actuators using the Yoshimura pattern with kinematic models demonstrating position accuracy of 1.27 mm and 0.373 mm in one direction under closed-loop control,^[9,10]^ respectively. However, none of their designs have been used to construct different robotic systems. W.-H. Chen et al. developed an algorithm for origami chains that automatically generate crease patterns,^[11]^ but their robots are built with thin materials, limiting their real-world applications, and the position accuracy of their modules has not yet been experimentally validated.

1. **2D Tessellation Generation**

Once $\kappa$ is chosen for the translational actuator, the crease parameter, $\mathcal{P}$, is determined following the procedure outlined in Algorithm 1. Taking $\boldsymbol{v}_{\boldsymbol{1,1}}$ as the reference vertex, then the coordinates of the remaining vertices are denoted as

$$\begin{aligned} \boldsymbol{v}_{i,j}=\boldsymbol{v}_{1,1}+\left[ \begin{matrix} \left( j-1 \right)H & \frac{\left( i-1 \right)H}{2} \end{matrix} \right]^{T}\#\text{(S1)} \end{aligned}$$

$$\begin{aligned} \boldsymbol{v}_{4,k}=\boldsymbol{v}_{1,1}+\left[ \begin{matrix} \left( j-0.5 \right)H & \frac{H}{2} \end{matrix} \right]^{T}\boldsymbol{\#}\text{(S2)} \end{aligned}$$

Algorithm 2 provides the computational steps for determining the origami structure: initialization of the actuator height, $H$, and the coordinate of the reference $\boldsymbol{v}_{1,1}$ in Line 1 and 2. Subsequent calculations to ascertain the coordinates of the vertices based on equation (S1) and (S2) are conducted in Line 3-10. The resulting coordinates are then compiled to form the $\mathcal{C}$ of the translational actuator. The forward kinematics model of the translational actuators can be expressed as

$$\begin{aligned} T_{trans}(d)=\left[ \begin{matrix} \begin{matrix} 1 & 0 & 0 & 0 \\ 0 & 1 & 0 & 0 \\ 0 & 0 & 1 & H-d \\ 0 & 0 & 0 & 1 \end{matrix} \end{matrix} \right]\#\text{(}\text{S3}\text{)} \end{aligned}$$

where $d\in[0, d_{c}]$ is the actual translational distance. Given the crease parameter for bending actuators, which include the maximal bending angle $\theta_{m}$ and the size factor $\kappa$, setting the specific parameters for these actuators is guided by Algorithm 3, utilizing the following equations,

$$\begin{aligned} \beta=\frac{1}{2}\theta_{m}\#\text{(S4)} \end{aligned}$$

$$\begin{aligned} r=\kappa\cot\beta\#\text{(S5)} \end{aligned}$$

$$\begin{aligned} L=2r\#\text{(S6)} \end{aligned}$$

$$\begin{aligned} H=4r\tan\beta\#\text{(S7)} \end{aligned}$$

$$\begin{aligned} c=\frac{H^{2}}{2L+2\sqrt{\left( \frac{1}{2}H \right)^{2}+L^{2}}}\#\text{(S8)} \end{aligned}$$

Analogs to the translational actuator, the 2D crease patterns ($\mathcal{C}$) coordinates for the bending actuator, relative to the initial vertex $\boldsymbol{v}_{1,1}$, can be computed based on

$$\begin{aligned} {\boldsymbol{v}_{i,j}=\boldsymbol{v}_{1,1}+\left[ \begin{matrix} \left( j//2 \right)L+\left( (j-1)//2 \right)H & \frac{\left( i-1 \right)H}{2} \end{matrix} \right]}^{T}\#\text{(S9)} \end{aligned}$$

$$\begin{aligned} \left\{ \begin{matrix} \boldsymbol{v}_{2,2}\text{ = }\boldsymbol{v}_{2,2}+\left[ \begin{matrix} c & 0 \end{matrix} \right]^{T} \\ \boldsymbol{v}_{2,3}=\boldsymbol{v}_{2,3}+\left[ \begin{matrix} -c & 0 \end{matrix} \right]^{T} \end{matrix} \right.\#\text{(S10)} \end{aligned}$$

To be highlighted, the central waterbomb unit in the bending module is defined by equation (S10). The generation of 2D crease pattern $\mathcal{C}$ is detailed in Algorithm 4, which begins by extracting the crease parameters, including actuator$H$, length $L$, and initializing the position of $\boldsymbol{v}_{1,1}$ in Line 1 and 2. Then, from Line 3 to 9, the coordinates of vertices are computed by employing equations (S9) and (S10). This structured geometrical modeling ensures that the crease pattern accurately conforms to the desired motion requirements. The forward kinematics model of the bending actuators can be expressed as

$$\begin{aligned} T_{bend}(\theta)=\left[ \begin{matrix} \begin{matrix} \cos\theta& 0 & \sin\theta& \mathcal{L}\sin(\vartheta)-\frac{L}{2} \\ 0 & 1 & 0 & 0 \\ -\sin\theta& 0 & \cos\theta& \mathcal{L}\cos(\vartheta)+\frac{H}{2} \\ 0 & 0 & 0 & 1 \end{matrix} \end{matrix} \right]\#\text{(}\text{S}\text{11)} \end{aligned}$$

where $\theta\in[0, \theta_{m}]$ is the actual bending angle, while $\vartheta=\theta+arctan(\frac{L}{H})$ and $\mathcal{L=}\frac{\sqrt{H^{2}+L^{2}}}{2}$ are for the simplification of expression.

1. **Kinematics Modelling with Thickness**

For the translational actuator, we account for the thickness error $t_{e}$ by adding it to the initial actuator height, thus compensating for the position error, which is given by

$$\begin{aligned} H^{*}=H+t_{e}\#\text{(S12)} \end{aligned}$$

The parameter $t_{e}$ takes into account both the material thickness and the width of the hinges, and is empirically determined from physical prototypes. Experimentation with our fabrication techniques has shown that $t_{e}$ is typically around 10 mm for translational actuators. The revised 2D crease pattern $\mathcal{C}^{\boldsymbol{*}}$ is then computed by introducing the new height $H^{*}$ from Algorithm 5 to recalculate the coordinates of the vertices.

Similarly, the motion range of the bending actuator is affected by material thickness, causing the folded structure to approximate a pentagonal shape instead of a quadrangle, thereby reducing the bending angle. Nevertheless, it is observed that the resulting pentagon can be inscribed within the intended quadrangle shape, as illustrated in **Figure 2**(d). The accommodated crease parameters $\mathcal{P}^{\boldsymbol{*}}$ for the bending actuator are calculated by

$$\begin{aligned} r_{e}=\frac{t_{e}}{2\sin\beta}\#\text{(S13)} \end{aligned}$$

$$\begin{aligned} r^{*}=r-r_{e}\#\text{(S14)} \end{aligned}$$

$$\begin{aligned} L^{*}=2r^{*}\#\text{(S15)} \end{aligned}$$

$$\begin{aligned} H^{*}=\frac{2r-r_{e}}{2r}H\#\text{(S16)} \end{aligned}$$

$$\begin{aligned} \beta^{*}=\tan^{-1} \frac{H^{*}}{{4r}^{*}}\#\text{(S17)} \end{aligned}$$

$$\begin{aligned} c^{*}=\frac{{H^{*}}^{2}}{2L^{*}+2\sqrt{\left( \frac{1}{2}H^{*} \right)^{2}+{L^{*}}^{2}}}\#\text{(S18)} \end{aligned}$$

Based on our characterization, $t_{e}$ is estimated to be around 4 mm in this case. The accommodated crease parameters $\mathcal{C}^{\boldsymbol{*}}$ are then computed by substituting the new $\mathcal{P}^{\boldsymbol{*}}$ values, calculated from Algorithm 6, into Algorithm 4. This thickness accommodation addresses material constraints by adjusting design parameters, improving the accuracy of motion and ensuring that the physical prototypes more closely match the intended theoretical models.

1. **Algorithm 1 to 6**

**Algorithm 1:**

**Algorithm 2:**

**
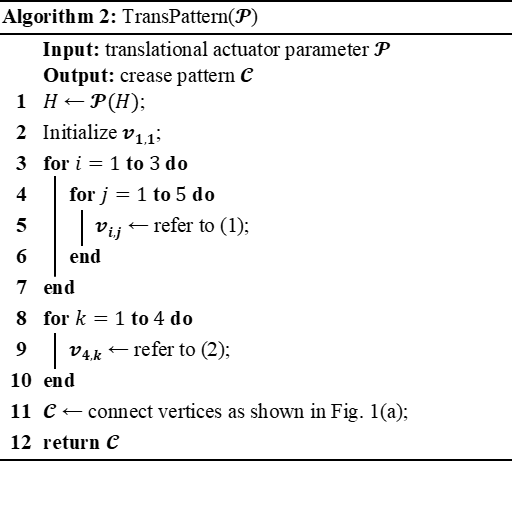
**

**Algorithm 3:**

**Algorithm 4:**

**
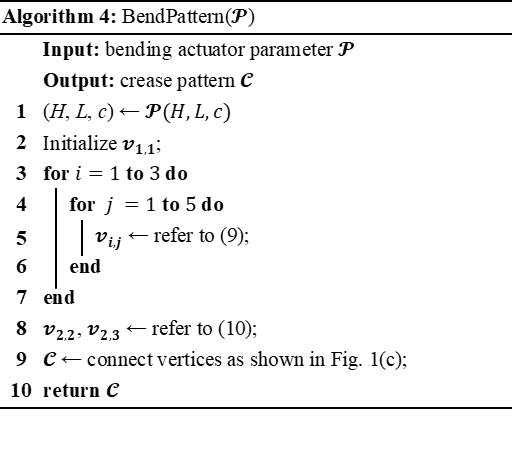
**

**Algorithm 5:**

**Algorithm 6:**

1. **Figure S1 to S3**


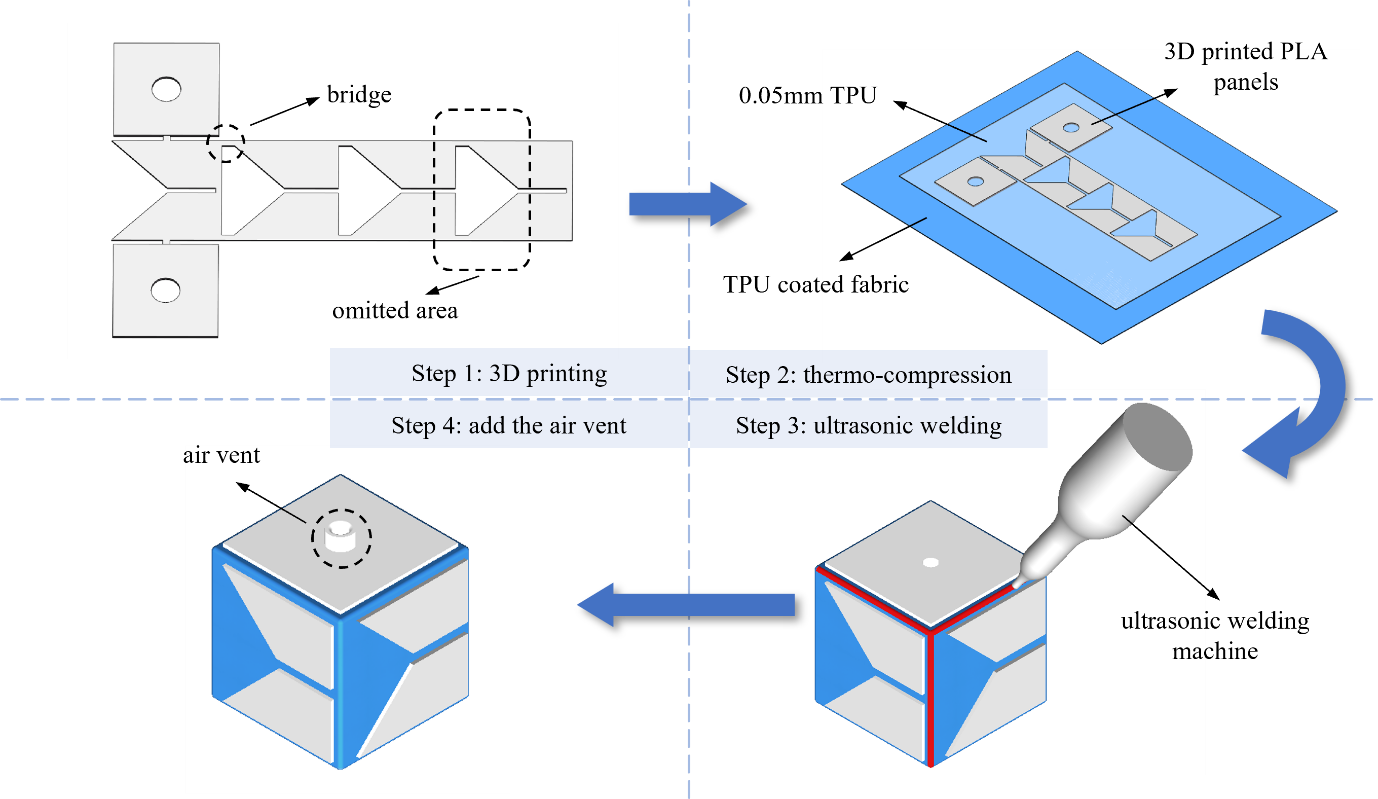


**Figure S1.** The fabrication process of the origami actuators. Step 1 shows the 3D printed PLA panels with connecting bridges. Step 2 illustrates the thermos-compression process. Step 3 uses an ultrasonic welding machine to seal the actuator. Step 4 shows a fabricated origami actuator with an air vent. The actuators are initialized at the undeformed state, requiring negative pressure to achieve the desired motions.


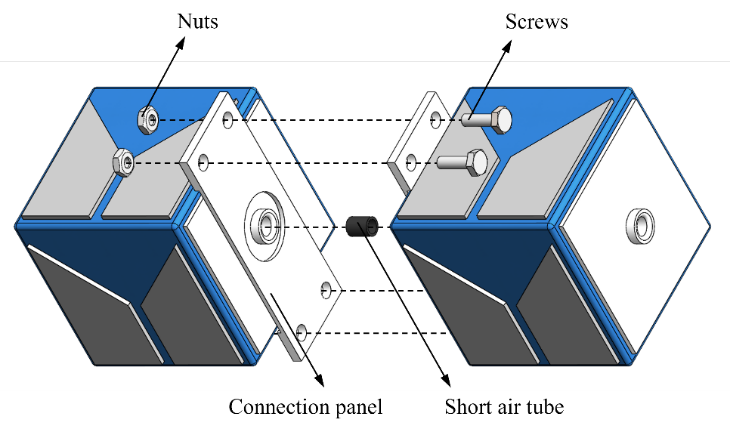


**Figure S2**. The connection of different LEGO-like origami actuators.

1. **Table S1 to S4**

**Table S1.** Comparison of The Key Aspects of Different Origami Actuators


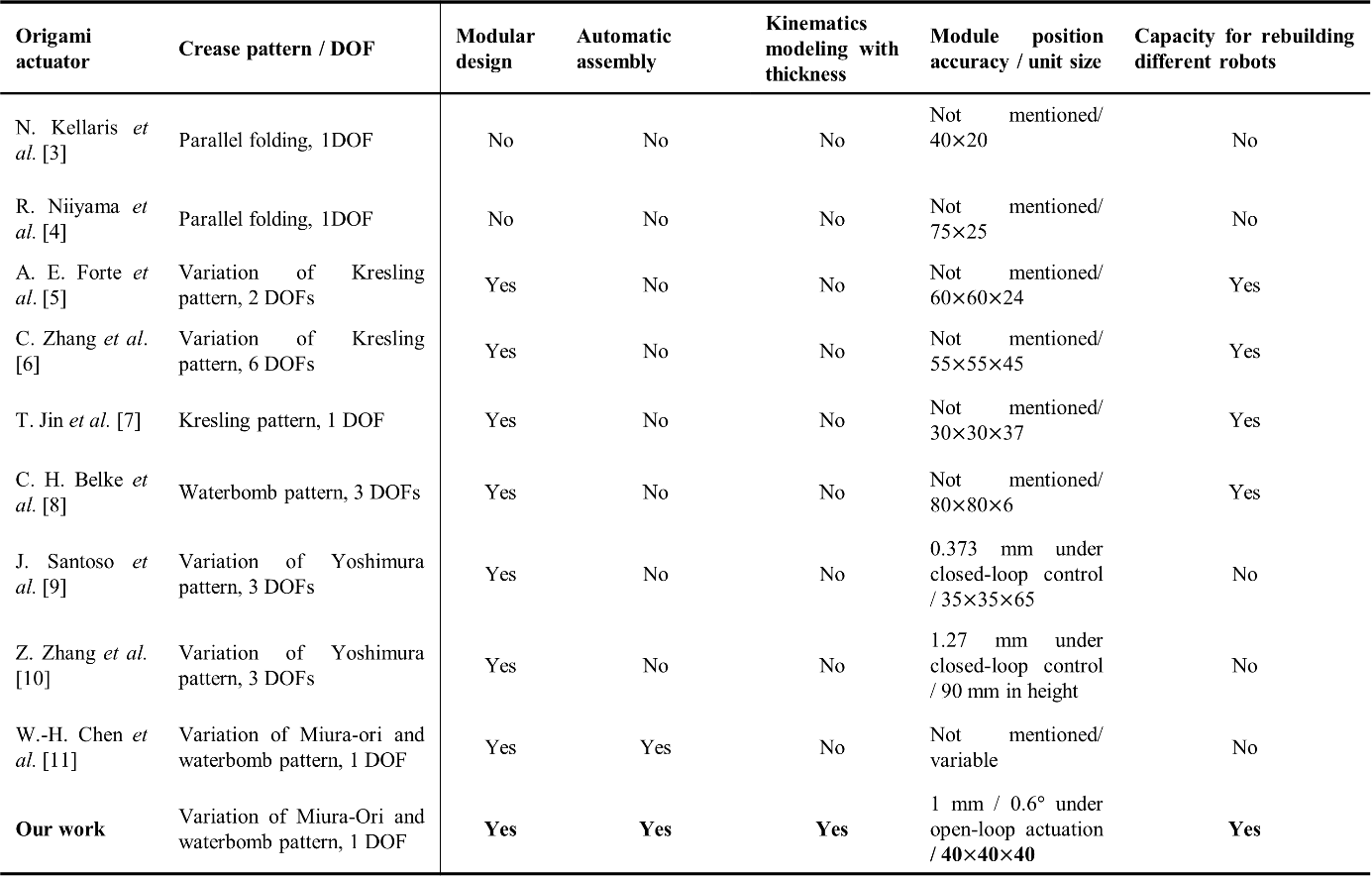


**Table S2.** Key Symbols and Their Descriptions


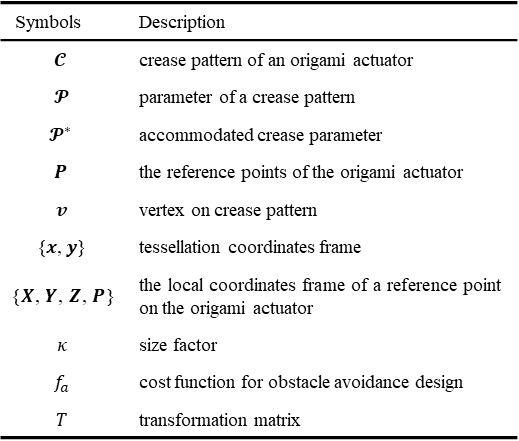


**Table S3.** The Crease Parameter under Normal Design and Thickness Accommodation Design

**
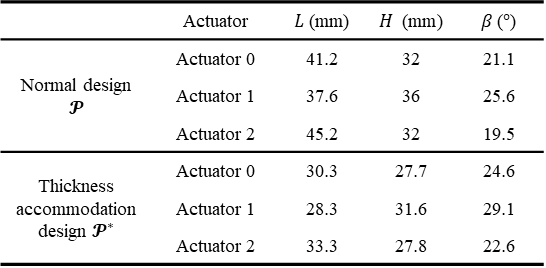
**

1. **Legends for Movie S1 to S3**

**Movie S1.**

The obstacle avoidance robot arm consists of four origami actuators and three passive links. When applying a negative pressure of -30 kPa, the robot arm successfully avoids the obstacle and place the toy hamburger in the plate.

**Movie S2.**

The sequential actuation tight space inspection robot combines both bending actuators and translational actuators. By sequentially providing negative pressure to the LEGO-like origami actuators, the robot can pass through the opening of a box and finish the inspection task.

**Movie S3.**

The bipedal walking robot consists of the bending actuators previously used in our tight space inspection robot. By applying positive and negative pressure alternatively, the biped robot walks approximately 240 mm within 10.4 s.

**References**

[1] Y. Chen, R. Peng, and Z. You, "Origami of thick panels," *Science,* vol. 349, no. 6246, pp. 396-400, 2015.

[2] R. Peng and G. S. Chirikjian, "Thick-panel origami structures forming seamless surfaces," *Nature Communications,* vol. 16, no. 1, p. 3881, 2025.

[3] N. Kellaris, V. Gopaluni Venkata, G. M. Smith, S. K. Mitchell, and C. Keplinger, "Peano-HASEL actuators: Muscle-mimetic, electrohydraulic transducers that linearly contract on activation," *Science Robotics,* vol. 3, no. 14, p. eaar3276, 2018.

[4] R. Niiyama, X. Sun, C. Sung, B. An, D. Rus, and S. Kim, "Pouch motors: Printable soft actuators integrated with computational design," *Soft Robotics,* vol. 2, no. 2, pp. 59-70, 2015.

[5] A. E. Forte, D. Melancon, L. M. Kamp, B. Gorissen, and K. Bertoldi, "MuA-Ori: Multimodal actuated origami," *arXiv preprint arXiv:2112.01366,* 2021.

[6] C. Zhang *et al.*, "Plug & play origami modules with all-purpose deformation modes," *Nature Communications,* vol. 14, no. 1, p. 4329, 2023.

[7] T. Jin, L. Li, T. Wang, G. Wang, J. Cai, Y. Tian, and Q. Zhang, "Origami-inspired soft actuators for stimulus perception and crawling robot applications," *IEEE Transactions on Robotics,* vol. 38, no. 2, pp. 748-764, 2021.

[8] C. H. Belke and J. Paik, "Mori: a modular origami robot," *IEEE/ASME Transactions on Mechatronics,* vol. 22, no. 5, pp. 2153-2164, 2017.

[9] J. Santoso, E. H. Skorina, M. Luo, R. Yan, and C. D. Onal, "Design and analysis of an origami continuum manipulation module with torsional strength," in *2017 IEEE/RSJ International Conference on Intelligent Robots and Systems (IROS)*, 2017: IEEE, pp. 2098-2104.

[10] Z. Zhang, G. Chen, Y. Xun, Y. Long, J. Wang, H. Wang, and J. Angeles, "Bioinspired rigid-soft hybrid origami actuator with controllable versatile motion and variable stiffness," *IEEE Transactions on Robotics,* 2023.

[11] W.-H. Chen, W. Yang, L. Peach, D. E. Koditschek, and C. R. Sung, "Kinegami: Algorithmic Design of Compliant Kinematic Chains From Tubular Origami," *IEEE Transactions on Robotics,* vol. 39, no. 2, pp. 1260-1280, 2022.
